# Supplementary material for: Drought recovery in plants triggers a cell-state-specific immune activation
Source: Nat Commun. 2025 Aug 29;16:8095. doi: 10.1038/s41467-025-63467-2 (PMC12397292; doi:10.1038/s41467-025-63467-2)
Supplement: Supplementary file 1 — Supplementary Information [file 41467_2025_63467_MOESM1_ESM.pdf]

Supplementary Information

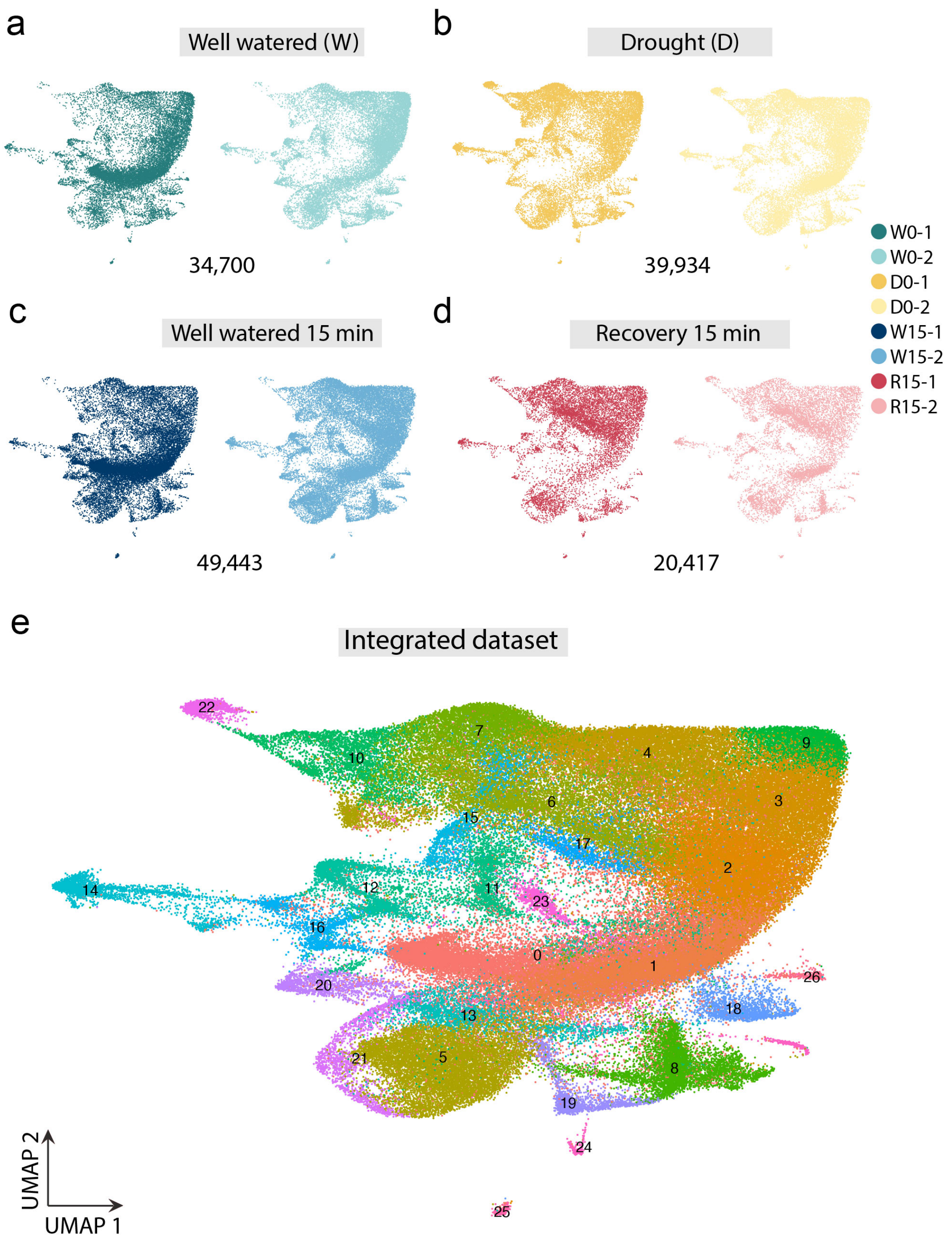

Supplementary Figure 1. Integration of single-nucleus RNA-seq data from biological replicates and treatment conditions identifies consistent cell identities.

a, UMAP projection of two independent replicates from well-watered plants (W0-1 and W0-2), showing 34,700 nuclei.
b, UMAP projection of drought-treated samples (D0-1 and D0-2), totaling 39,934 nuclei.
c, UMAP projection of samples collected 15 minutes after rehydration of well-watered plants (W15-1 and W15-2), with 49,443 nuclei.
d, UMAP projection of samples collected 15 minutes after rehydration of drought-treated plants (R15-1 and R15-2), with 20,417 nuclei.
e, Integrated UMAP of all samples colored by Seurat cluster assignment, identifying 27 transcriptionally distinct clusters. Cells from all treatment groups are well-mixed across clusters, indicating robust integration of biological replicates and treatment conditions.

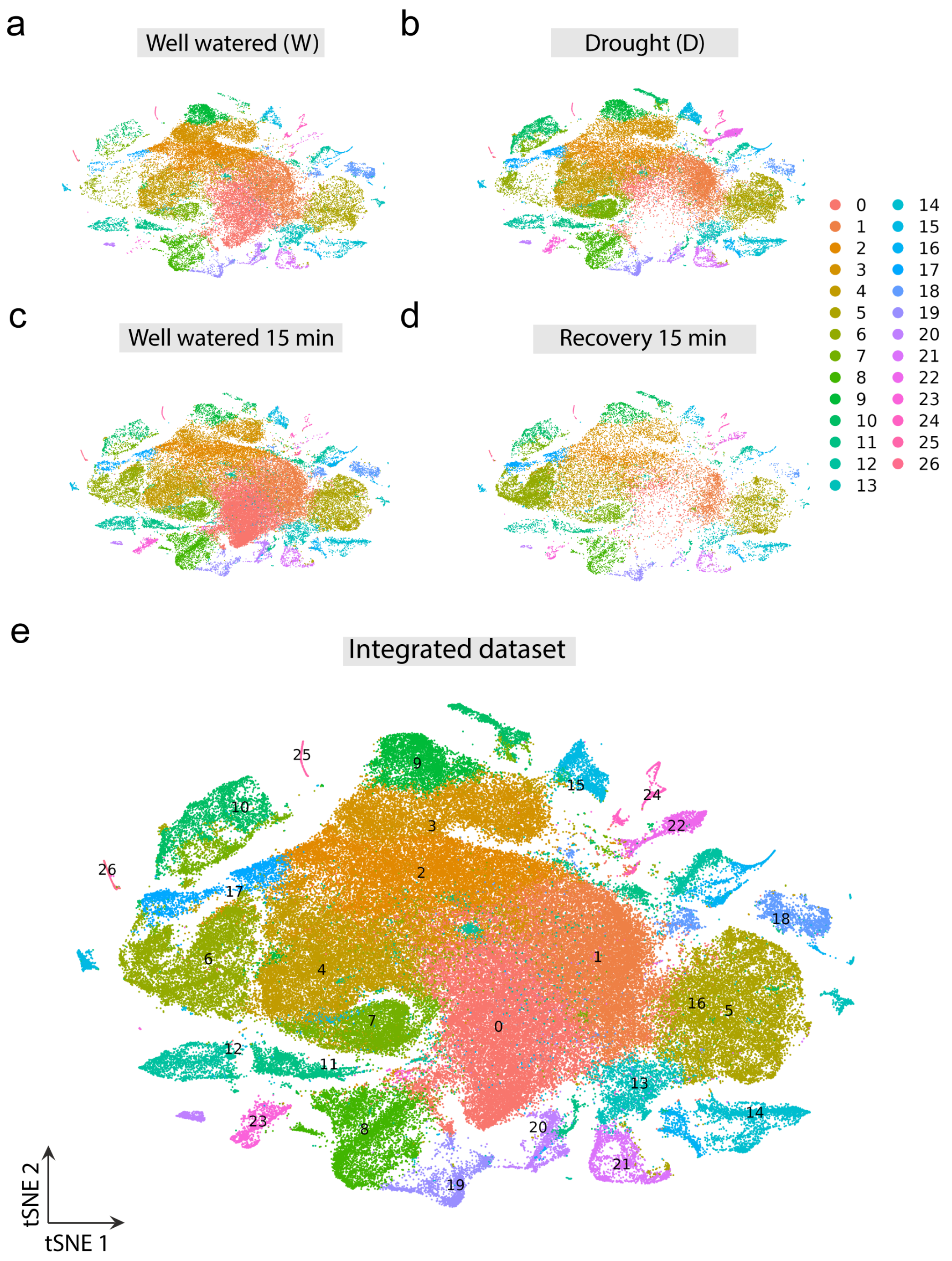

Supplementary Figure 2. tSNE projection confirms robust integration of single-nucleus transcriptomes across conditions.

a, tSNE projection of well-watered samples (W), showing clustering of major cell types with minimal batch separation.
b, tSNE projection of drought-treated samples (D), illustrating preservation of cell-type structure under drought.
c, tSNE projection of samples collected 15 minutes after rehydration from well-watered conditions, showing similar clustering patterns to baseline.
d, tSNE projection of samples collected 15 minutes after rehydration from drought conditions (Recovery 15 min), indicating maintenance of distinct cell identities.
e, Integrated tSNE map of all samples colored by Seurat cluster (0–26), demonstrating consistent clustering across all conditions and replicates. Cell clusters are well-integrated and distributed, indicating successful batch correction and integration.

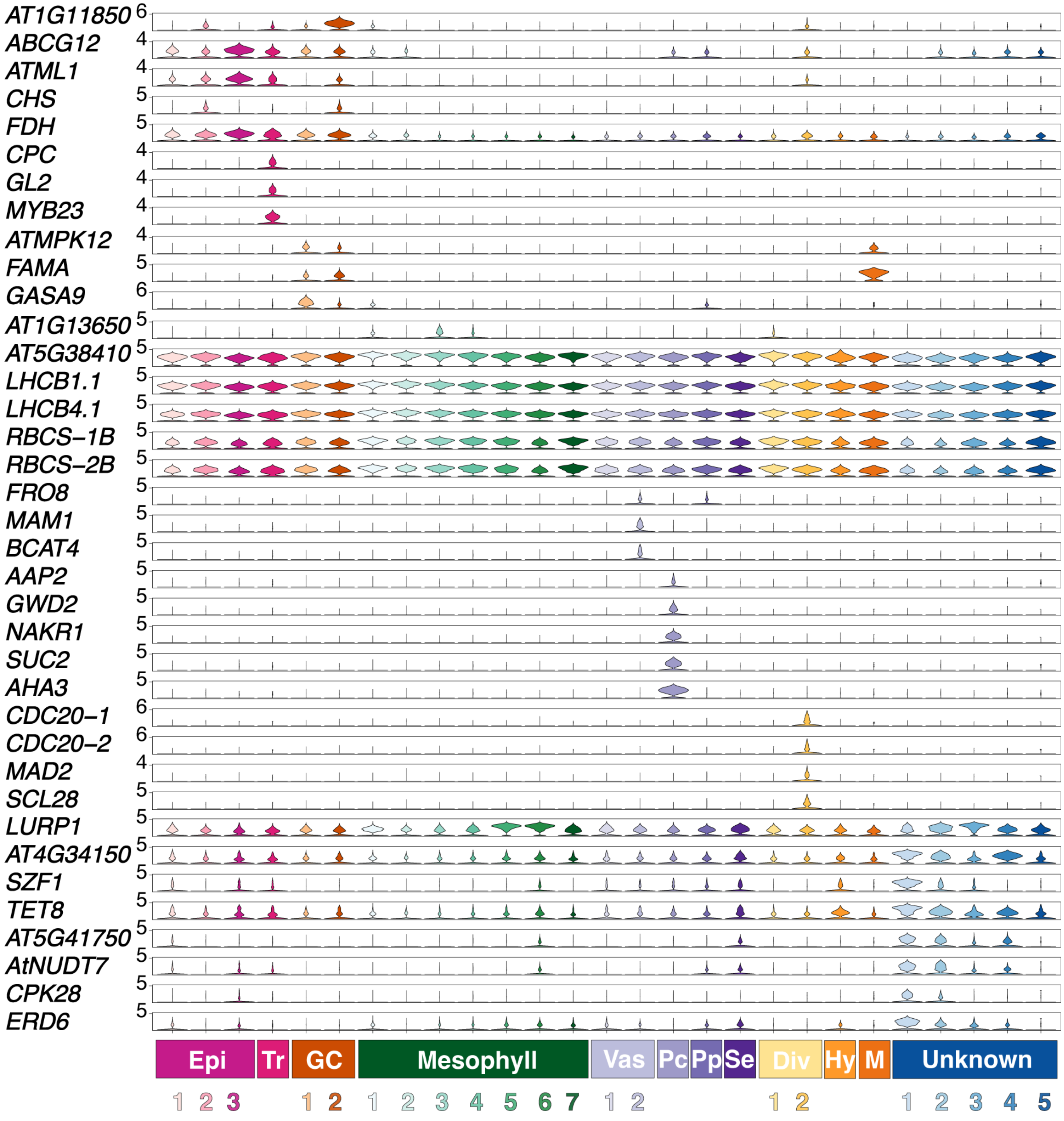

Supplementary Figure 3. Expression of known tissue- and cell-type–specific marker genes across clusters.

Violin plots display the expression of literature-curated marker genes across 27 annotated clusters in the integrated dataset, corresponding to major leaf cell types. Cell types are color-coded and include: **Epi** (Epidermal; clusters 1–3), **Tr** (Trichome), **GC** (Guard Cell; clusters 1-2), **Mesophyll** (clusters 1–7), **Vas** (Vascular; clusters 1-2), **Pc** (Phloem companion), **Pp** (Phloem parenchyma), **Se** (Sieve element), **Div** (Dividing cells; 1–2), **Hy** (Hydathode), **M** (Myrosin cells), and **Unknown** (clusters 1–5). Violin width represents cell proportion expressing the gene.

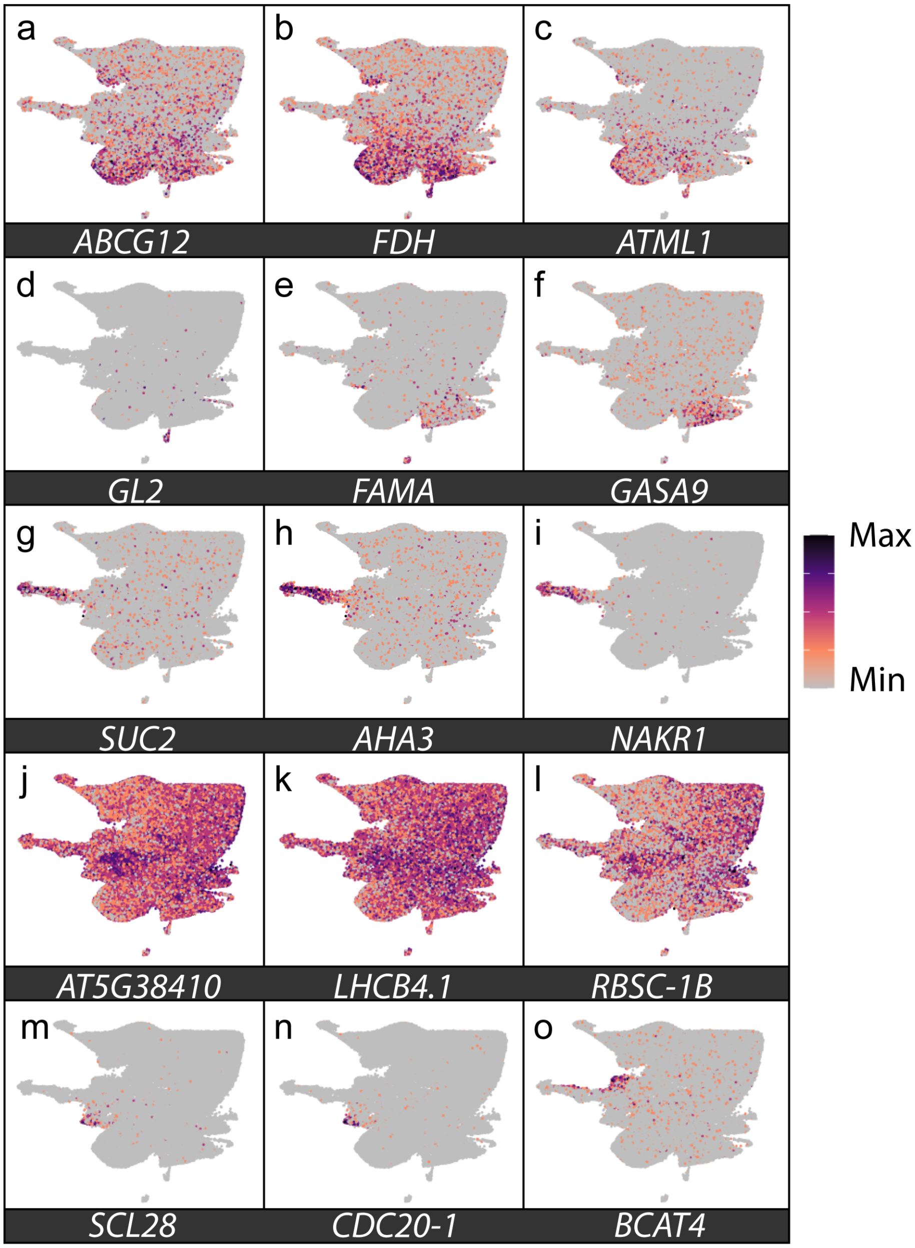

Supplementary Figure 4. Spatial projection of literature-based marker genes across the integrated UMAP.

a, *ARABIDOPSIS THALIANA WHITE-BROWN COMPLEX 12* (*ABCG12*), encodes an ABC transporter involved in cuticular wax biosynthesis.
b, *FIDDLEHEAD* (*FDH*), an epidermis-specific gene, encodes KCS10, a putative 3-ketoacyl-CoA synthase.
c, *MERISTEM LAYER 1* (*ATML1*), epidermis specific, encodes a homeobox protein similar to GL2.
d, *GLABRA 2* (*GL2*) a homeodomain protein that affects epidermal cell identity, including trichomes.
e, *FAMA* (*FMA*), Encodes a basic helix-loop-helix transcription factor whose activity is required to promote differentiation of stomatal guard cells and to halt proliferative divisions in their immediate precursors.
f, *GIBBERELLIC ACID STIMULATED ARABIDOPSIS 9* (*GASA9*).
g, *SUCROSE-PROTON SYMPORTER 2* (*SUC2*), Encodes for a high-affinity transporter essential for phloem loading and long-distance transport. A major sucrose transporter.
h, *Arabidopsis H(+)-ATPase isoform 3* (*AHA3*)**.**
i, *SODIUM POTASSIUM ROOT DEFECTIVE 1* (*NaKR1*) encodes a phloem mobile metal binding protein necessary for phloem function and root meristem maintenance.
j, *RUBISCO SMALL SUBUNIT 3B* (*RBCS3B*), encodes a member of the Rubisco small subunit (RBCS) multigene family.
k, *LIGHT HARVESTING COMPLEX PHOTOSYSTEM II* (*LHCB4.1*).

l, *RUBISCO SMALL SUBUNIT 1B* (*RBCS1B*)**.**
m, *SCARECROW-LIKE 28* (*SCL28*) transcription factor belonging to the GRAS family which controls the mitotic cell cycle and division plane orientation.
n, *CELL DIVISION CYCLE 20.1* (*CDC20.1*) Encodes a CDC20 protein that interacts with APC subunits, components of the mitochondrial checkpoint complex and mitotic cyclin substrates and is indispensable for normal plant development and fertility.
o, *BRANCHED-CHAIN AMINOTRANSFERASE 4* (*BCAT4*) belongs to the branched-chain amino acid aminotransferase gene family. Encodes a methionine-oxo-acid transaminase.

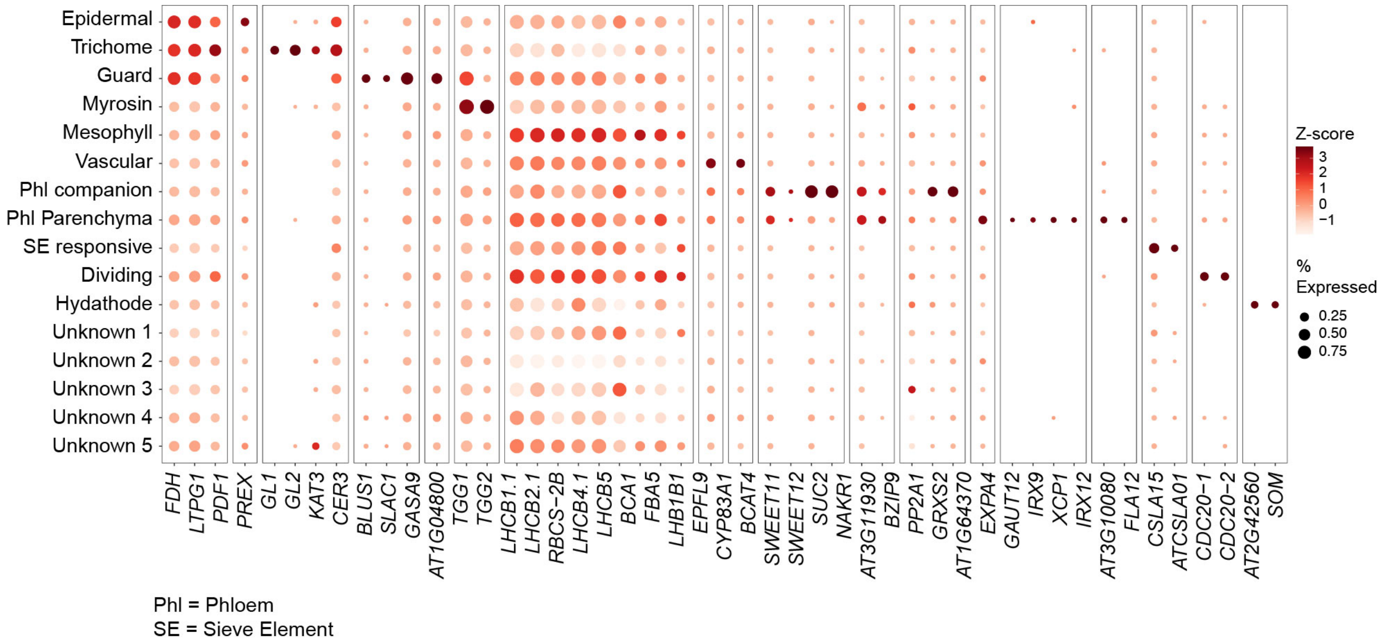

Supplementary Figure 5. Expression of marker genes across annotated leaf cell types in Arabidopsis.

Dot plot showing the expression patterns of selected marker genes across various annotated cell types in the leaf. Each row represents a cell type, and each column corresponds to a marker gene. Dot size indicates the percentage of cells expressing the gene within the respective cell type, while dot color represents the scaled expression level (Z-score), with deeper red indicating higher expression. Phloem-associated clusters, including phloem companion, phloem parenchyma, and sieve element (SE) responsive cells, show distinct expression of canonical genes such as *SWEET11*, *SUC2*, and *PP2-A1*. Other clusters such as mesophyll, guard, epidermal, and unknown populations exhibit distinct transcriptional signatures, including photosynthetic genes (*RBCS2B*, *LHCBs*) and stress-responsive genes (*PDF1*, *PRX*). Phl = Phloem; SE = Sieve Element. Color scale represents normalized expression intensity from low (light) to high (dark). Gray cells indicate no detectable expression.

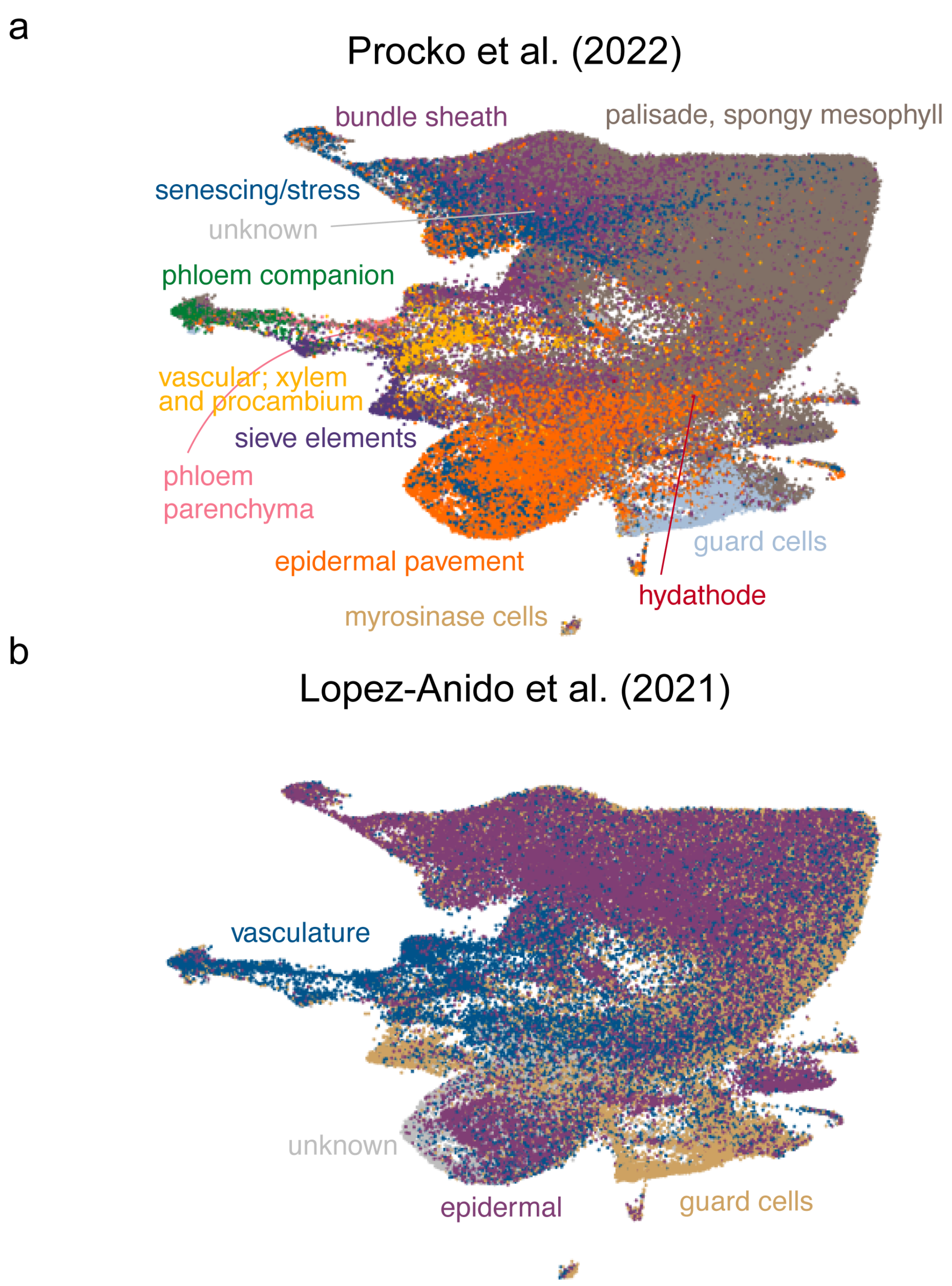

Supplementary Figure 6. Projection of publicly available annotated single-cell Arabidopsis leaf datasets onto our Arabidopsis rosette single-nucleus transcriptome.

a, Cell type annotations from Procko et al. (2022) projected onto the integrated UMAP of our single-nucleus RNA-seq dataset. Distinct cell types—including epidermal pavement, guard cells, mesophyll, bundle sheath, hydathode, phloem parenchyma, phloem companion, sieve elements, vascular (xylem/procambium), myrosinase cells, and senescing/stress cells—show strong overlap with transcriptional clusters in our dataset.

b, Cell type annotations from Lopez-Anido et al. (2021) projected onto the same UMAP. Major cell identities—including vascular, epidermal, and guard cells—align well with our transcriptional clusters, providing external validation for our cell type assignments.

Together, these reference-based projections confirm the robustness of our integrated dataset and support the annotation of less well-characterized populations.

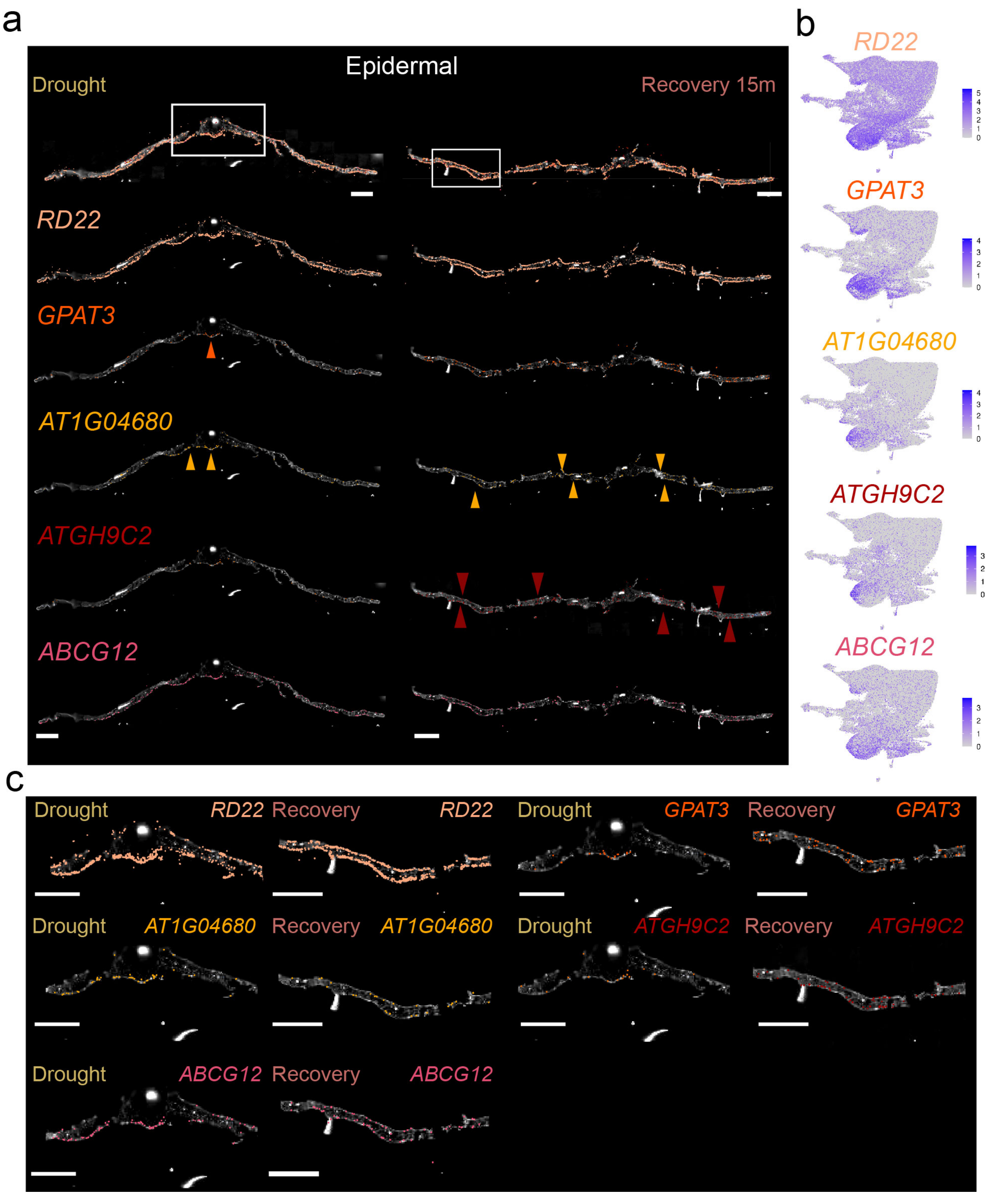

Supplementary Figure 7. Spatial expression dynamics of epidermal genes during drought and early recovery in *Arabidopsis*.

a, Spatial transcriptomics maps of five epidermal genes under drought (left) and 15 minutes post-rewatering (recovery; right) conditions. Each row corresponds to one gene: *RD22*, *GPAT3*, *AT1G04680*, *ATGH9C2*, and *ABCG12*. The merged panels overlay gene expression onto tissue structure. Colored arrowheads highlight regions of enriched expression: orange for *GPAT3*, yellow for *AT1G04680*, and red for *ATGH9C2*. White boxes indicate regions shown in panel **c,** Scale bars = 1 mm.
b, UMAP projections of the entire tissue for each gene, showing normalized (log-transformed) expression levels. Color intensity indicates expression from low (blue) to high (white).

c, Magnified views of boxed regions from panel a, comparing spatial expression between drought and recovery conditions for each gene. These views reveal both stable and dynamic gene expression changes. Scale bars = 1 mm.

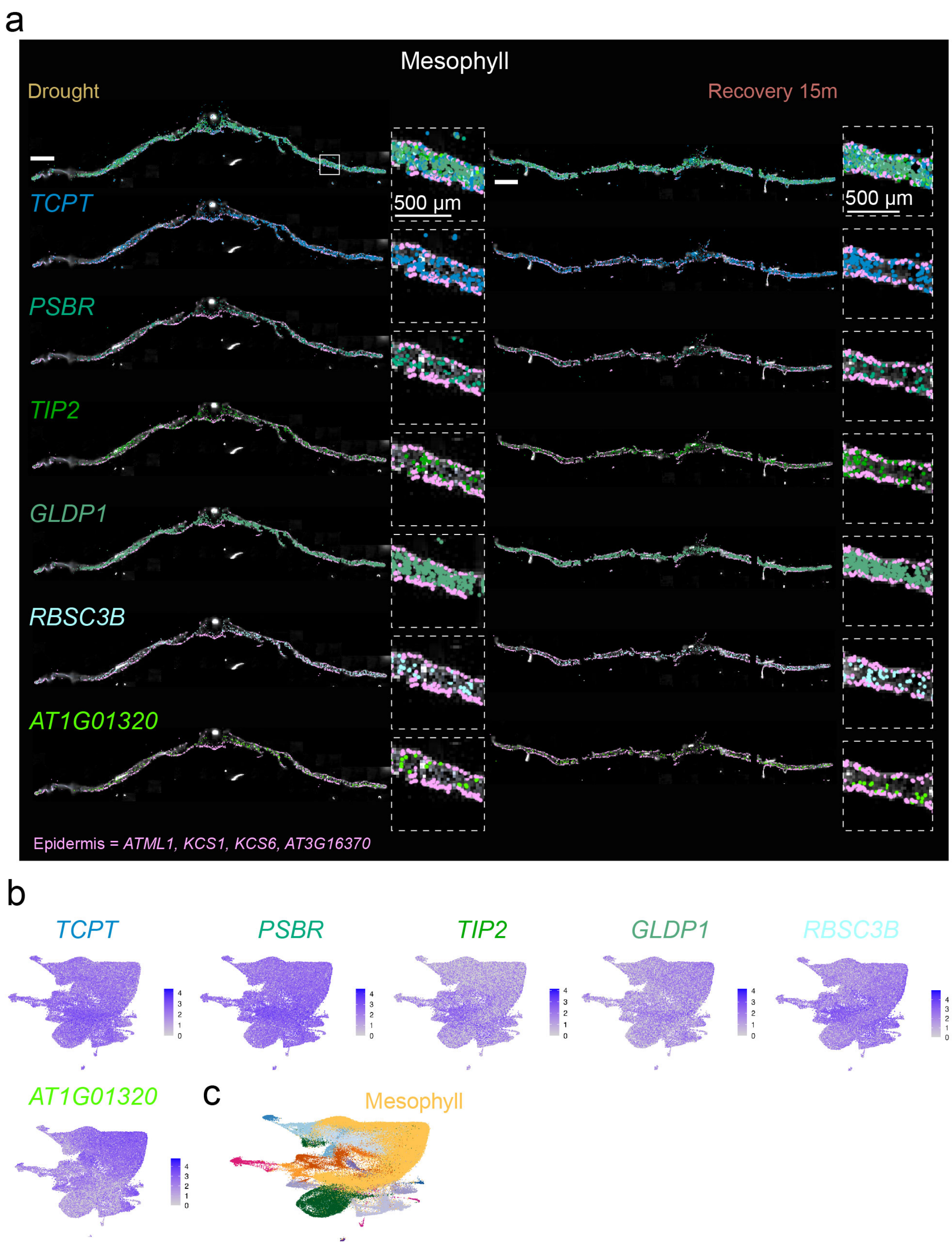

Supplementary Figure 8. Spatial expression of mesophyll-associated genes during drought stress and early recovery in Arabidopsis.

a, Spatial maps showing expression of mesophyll marker genes under drought (left panels) and 15-minute rewatering recovery (right panels). Expression of six genes is shown: *TCPT*, *PSBR*, *TIP2*, *GLDP1*, *RBSC3B*, and *AT1G01320*. Merged images display gene expression overlayed on tissue structure, with epidermal signal in pink and mesophyll signal in green. Insets (dashed boxes) highlight a zoomed-in mesophyll region under each condition to illustrate cell-type–specific spatial expression. Scale bars = 1 mm (unless indicated otherwise).
b, UMAP projections of whole-tissue transcriptomes showing normalized expression (log scale) for each mesophyll marker gene. Blue-to-white color gradient indicates low to high expression. Genes shown include: *TCPT*, *PSBR*, *TIP2*, *GLDP1*, *RBSC3B*, and *AT1G01320*.

c, UMAP embedding of all cells, colored by annotated cell type, highlights the mesophyll cluster in orange. This reference map aids interpretation of gene localization shown in b and a.

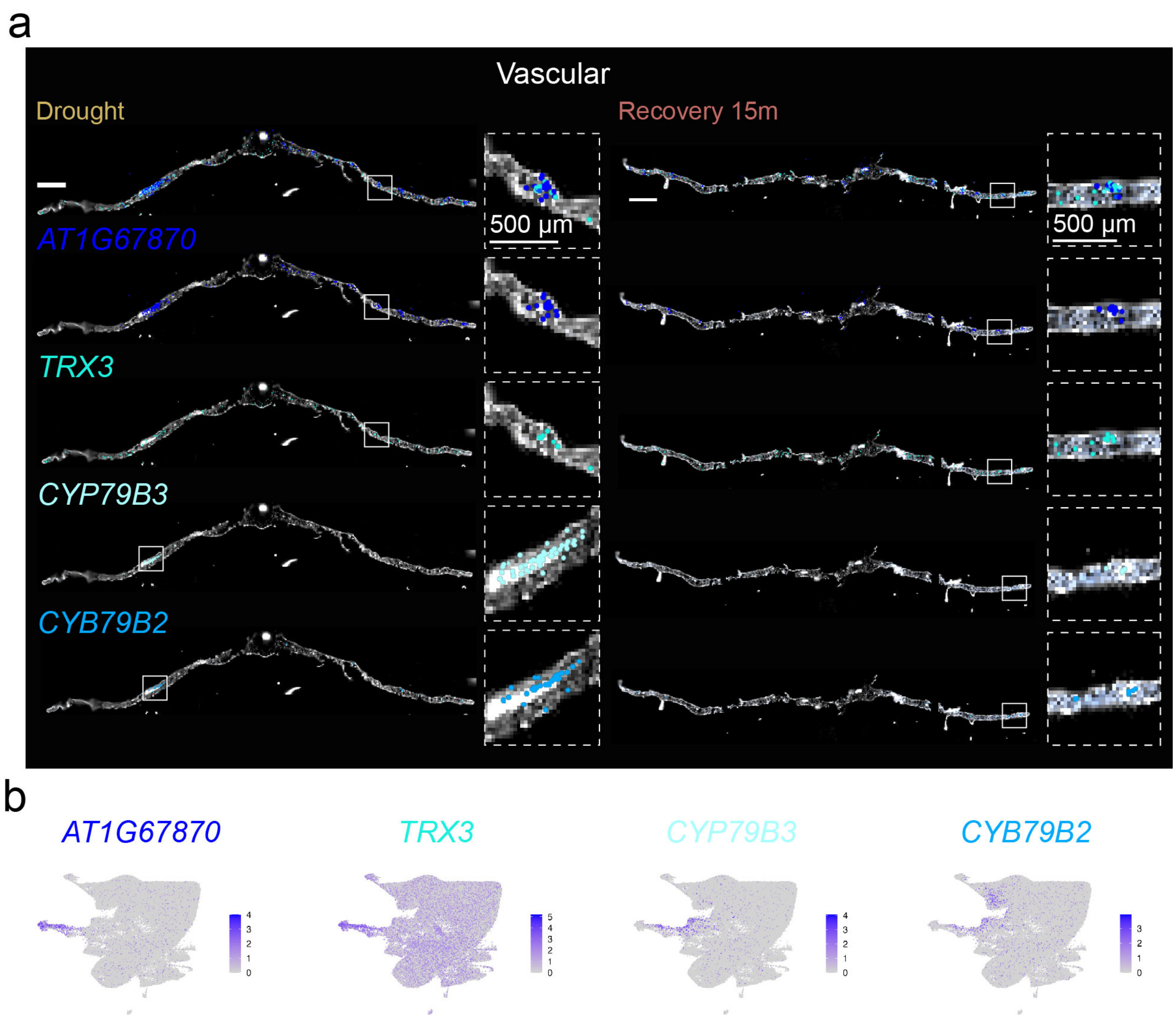

Supplementary Figure 9. Spatial expression of vasculature-localized transcripts during drought stress and early recovery in Arabidopsis.

a, Spatial transcriptomic maps showing vascular marker gene expression under drought (left) and 15 minutes after rewatering (recovery, right). Rows represent individual genes: *AT1G67870*, *TRX3*, *CYP79B3*, and *CYB79B2*. Merged panels overlay gene expression on anatomical structure. White arrows highlight vascular strands; white boxes outline zoom-in regions shown in adjacent insets. Insets reveal localized expression patterns within vascular bundles. Scale bars = 1 mm (unless indicated otherwise).

b, UMAP projections of all profiled cells displaying normalized expression (log-transformed) for each vascular gene, colored from low (blue) to high (white). Each gene shows restricted expression in vascular-associated clusters, consistent with spatial localization observed in panel a.

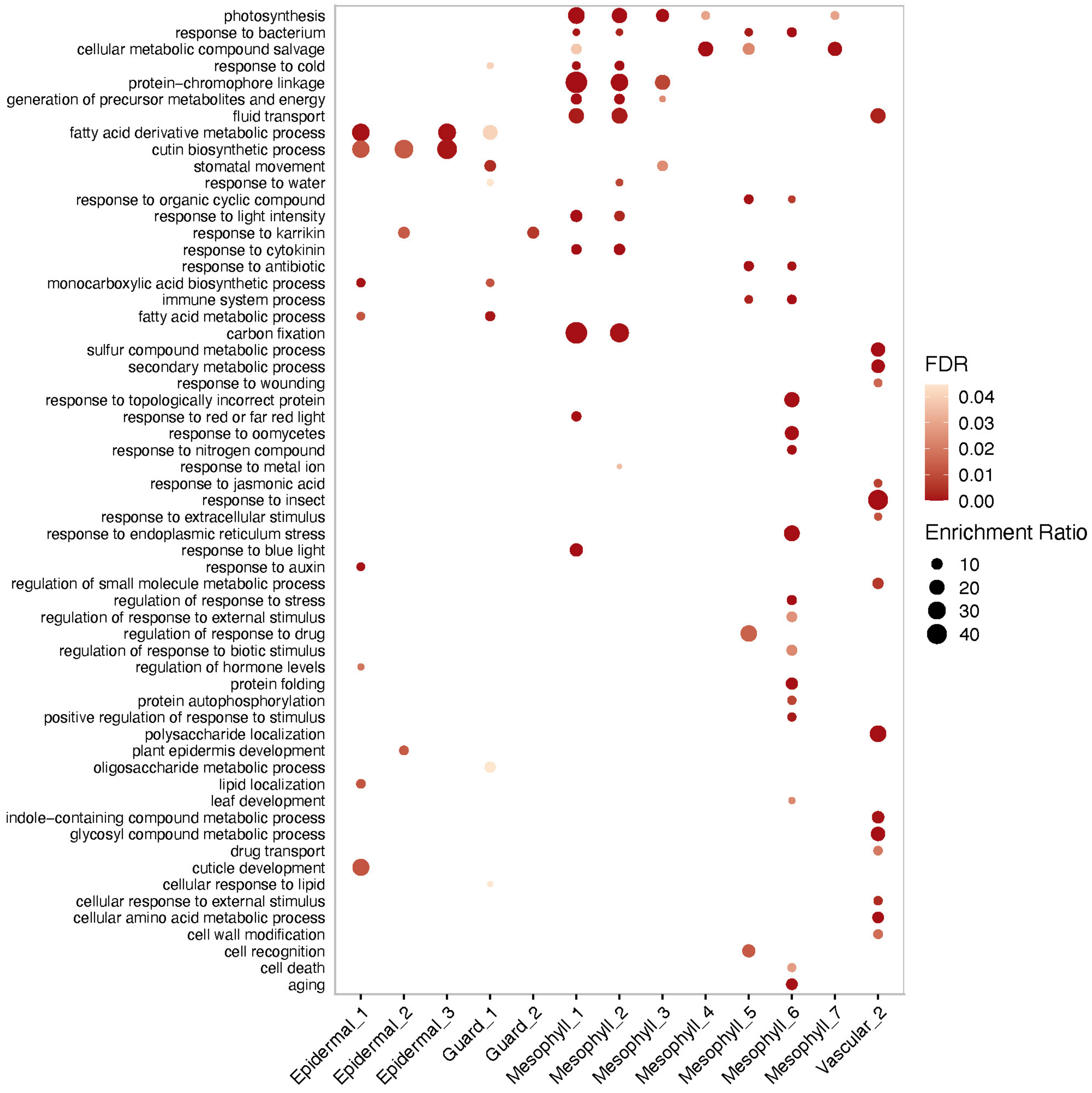

Supplementary Figure 10. GO term enrichment analysis across leaf cell types.

Dot plot showing Gene Ontology (GO) enrichment for biological processes across annotated cell types in the leaf. Each row represents a GO term, and each column corresponds to a specific cell type. Dot color reflects the false discovery rate (FDR), with darker red indicating more significant enrichment. Dot size corresponds to the enrichment ratio, indicating the degree of overrepresentation of each GO term in the respective cell type.

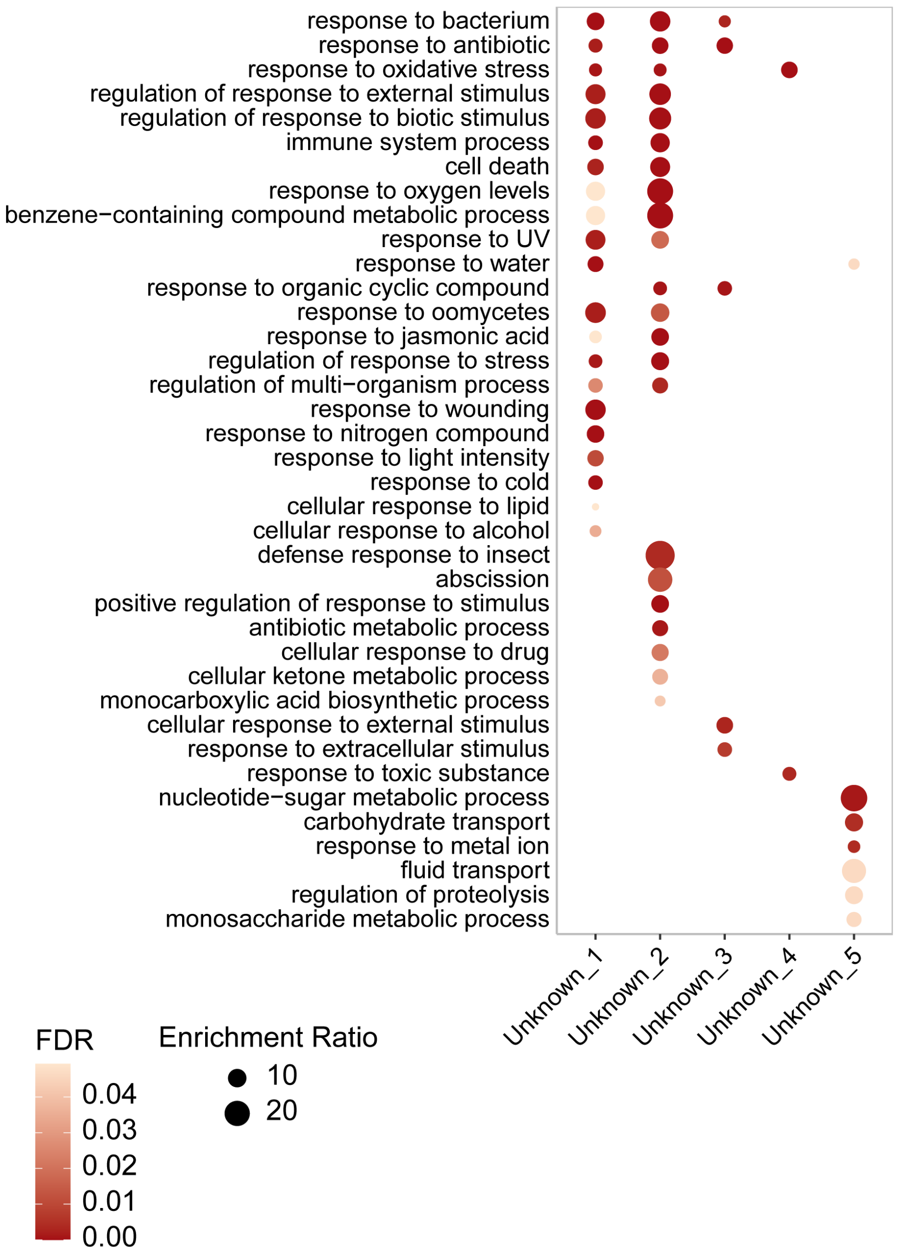

Supplementary Figure 11. Gene Ontology (GO) enrichment analysis of five unannotated clusters.

GO enrichment analysis was performed on five clusters that remained unannotated following initial classification. Each dot represents a significantly enriched GO term, with color indicating the false discovery rate (FDR) and size indicating the enrichment ratio. Notably, Cluster Unknown 5 shows strong enrichment in nucleotide-sugar metabolic processes, potentially reflecting changes in glycosylation, cell wall biosynthesis, or other nucleotide-sugar-dependent pathways[101]. This cluster also displays enrichment for carbohydrate transport, suggesting altered regulation of sugar movement across membranes and shifts in energy metabolism or nutrient uptake[102]. Dot size corresponds to the enrichment ratio, indicating the degree of overrepresentation of each GO term in the respective cell type.

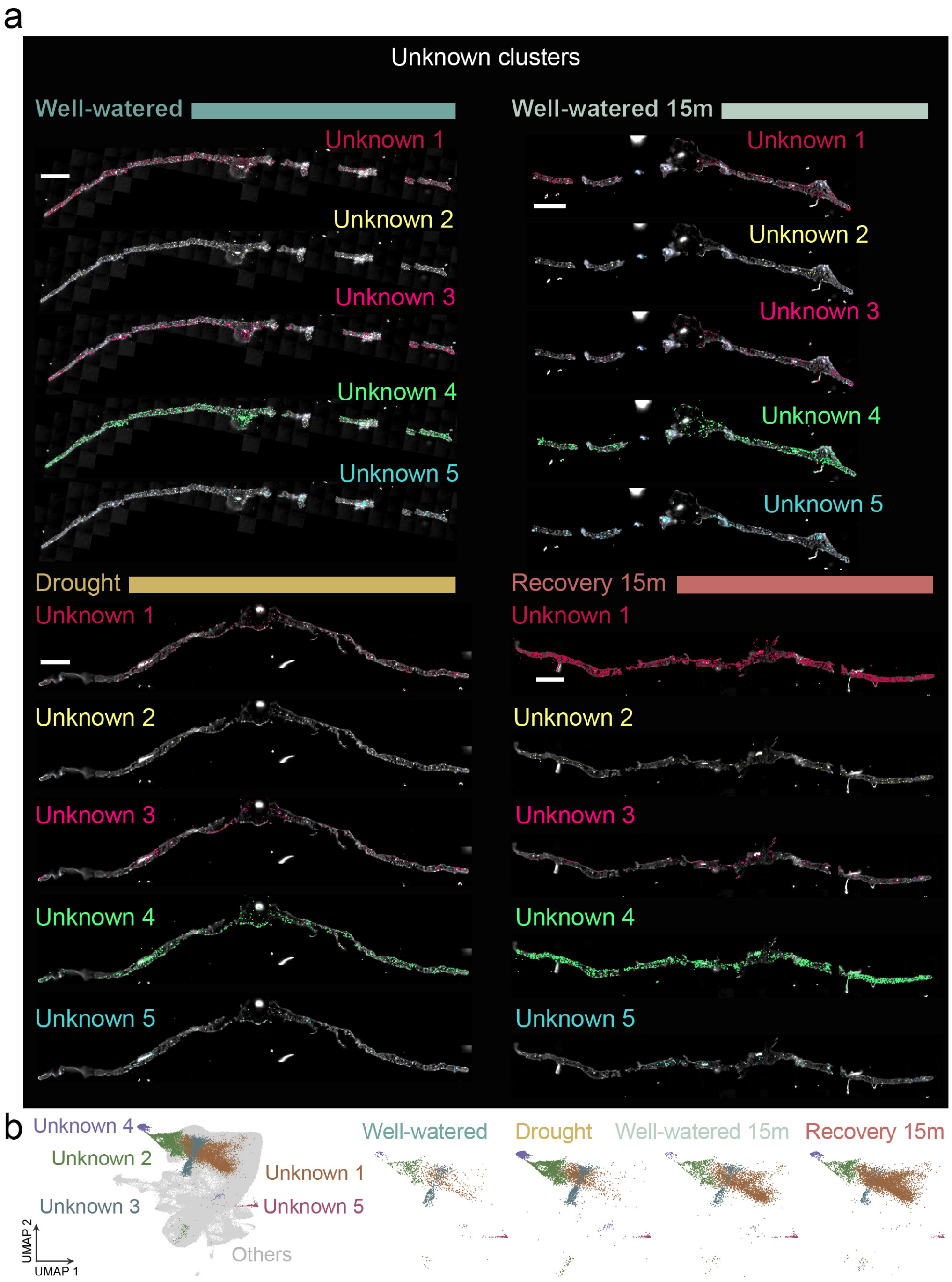

Supplementary Figure 12. Spatial distribution and dynamics of uncharacterized cell clusters across water status conditions.

a, Spatial transcriptomic maps showing the localization of five uncharacterized cell clusters (Unknown 1–5) across four treatment conditions: well-watered (top left), drought (bottom left), 15 minutes after rewatering from well-watered (top right), and 15 minutes after rewatering from drought (bottom right). Each row represents one unknown cluster, colored uniquely (Unknown 1–5) and overlaid on tissue sections to visualize their spatial enrichment. Differences in localization and intensity across conditions suggest distinct functional or developmental identities. Scale bars = 1 mm.
b, UMAP projections of single-cell transcriptomes highlighting the five unknown clusters (left) and their distribution across treatment conditions (right). Clusters are colored consistently with panel a**,** Right panel shows each treatment condition separately to illustrate shifts in cell-type proportions and state under drought and recovery.

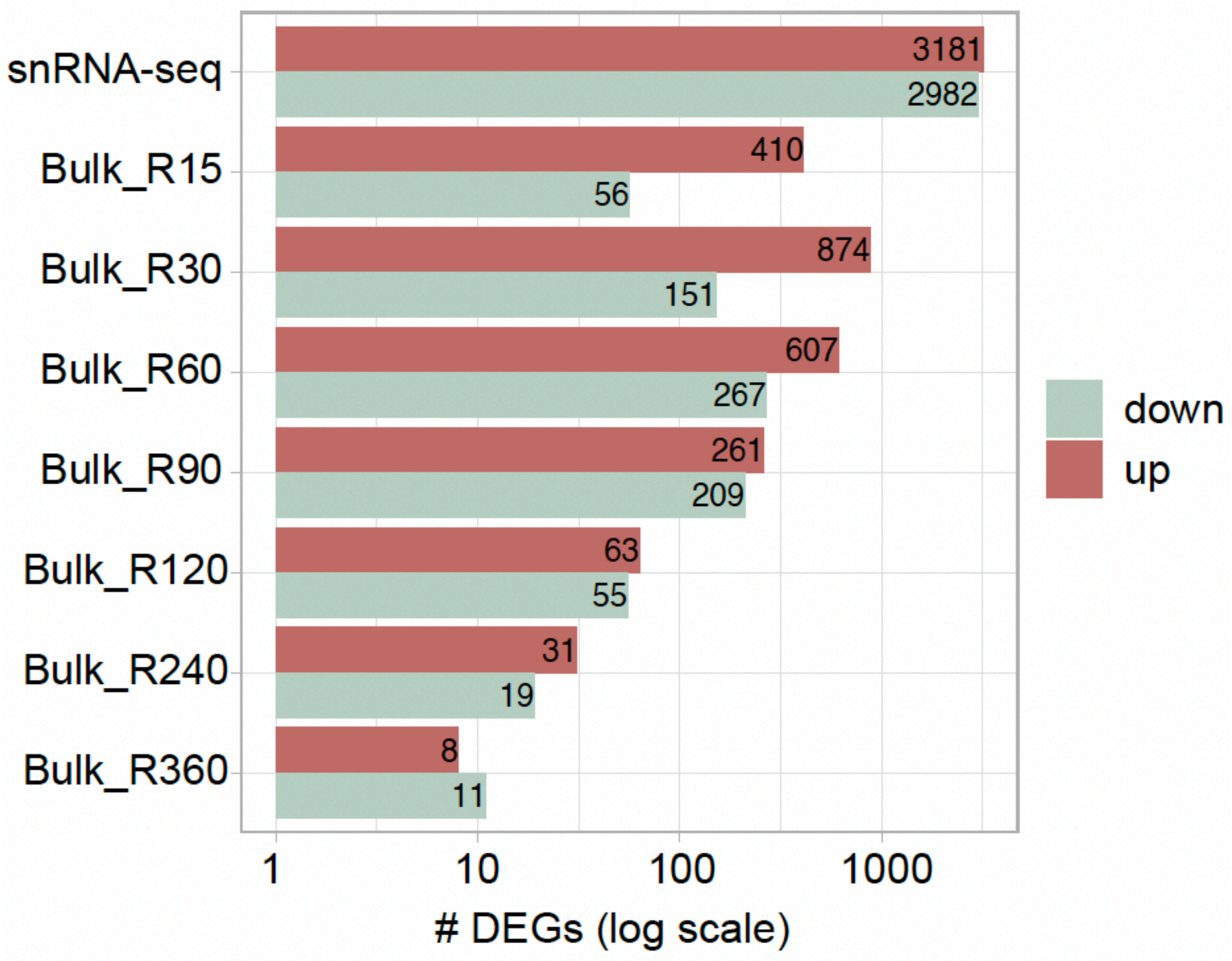

Supplementary Figure 13. Single-nucleus RNA-seq reveals a substantially higher number of differentially expressed genes (DEGs) during early recovery compared to bulk RNA-seq.

Bar plot showing the number of significantly upregulated (red) and downregulated (green) DEGs at various time points following rehydration after drought stress. The x-axis represents the number of DEGs on a log scale. The snRNA-seq dataset (top) corresponds to 15 minutes post-rehydration and reveals over 6,000 DEGs–approximately 10-fold more than detected by bulk RNA-seq at the same time point (Bulk R15).

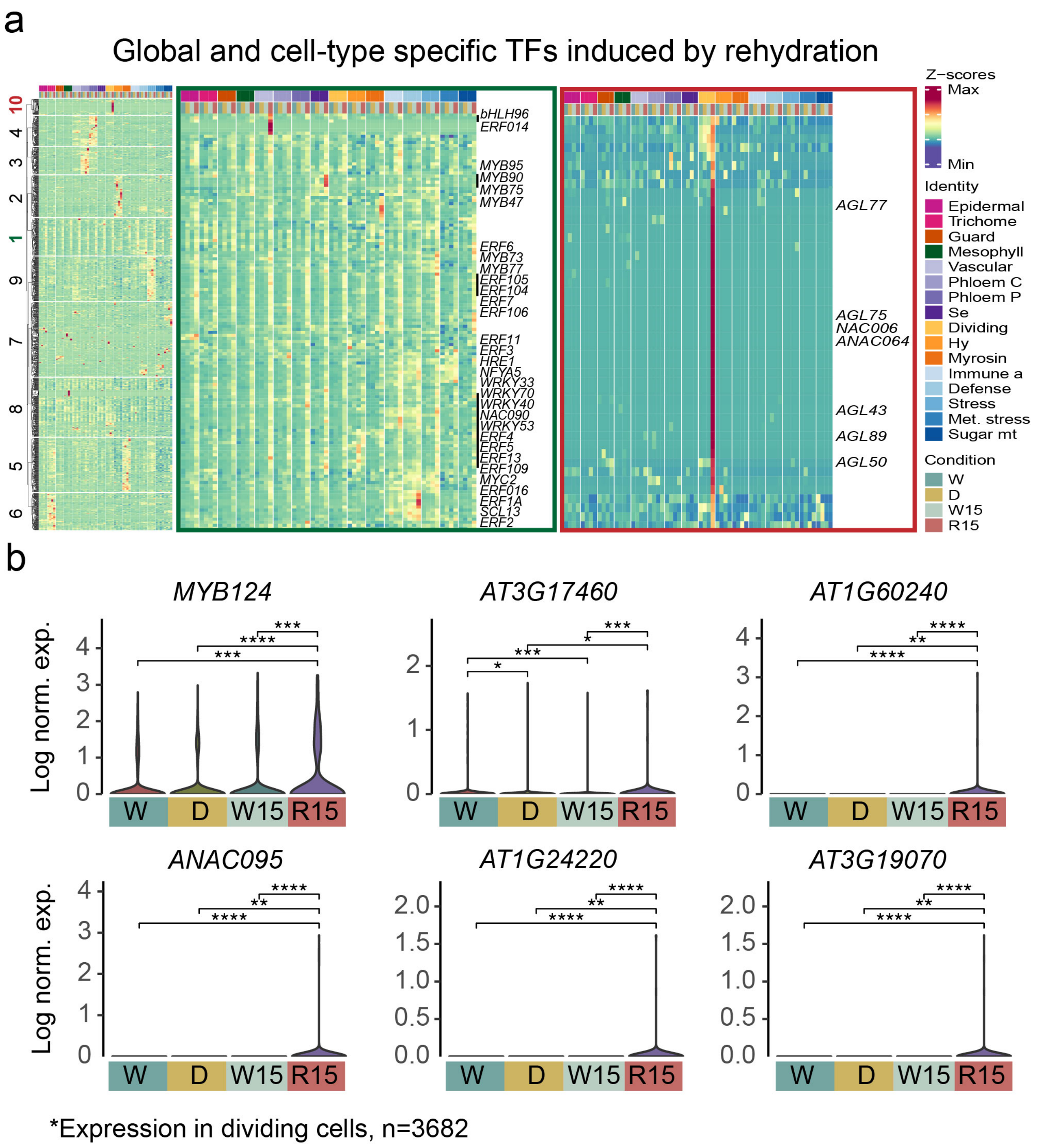

Supplementary Figure 14. Transcription factors induced by rehydration, including global and cell-type–specific responses.

a, Heatmap showing transcription factor (TF) expression dynamics across all annotated cell types under different water conditions. Rows represent individual TF genes, grouped into 10 hierarchical clusters (left bar), and columns represent single cells, colored by cell identity (top) and treatment condition (bottom: W = well-watered, D = drought, W15 = 15 min post-watering from well-watered, R15 = 15 min post-rehydration from drought). Two groups of rehydration-induced TFs are highlighted: globally induced TFs (green box) and cell-type–specific TFs enriched particularly in dividing cells (red box). Selected TFs are labeled, including *MYB124/FLP*, *ERF014*, *NAC006*, and *AGL77*.
b, Violin plots showing log-normalized expression of six representative rehydration-induced TFs—*MYB124*, *AT3G17460*, *AT1G60240*, *ANAC095*, *AT1G24220*, and *AT3G19070*—across the four conditions in dividing cells (n = 3,682). Statistical comparisons between conditions (pairwise Wilcoxon test) reveal significant rehydration-

induced upregulation in R15 compared to other states. Significance levels: *p* < 0.05 (*), < 0.01 (**), < 0.001 (***), and < 0.0001 (****).

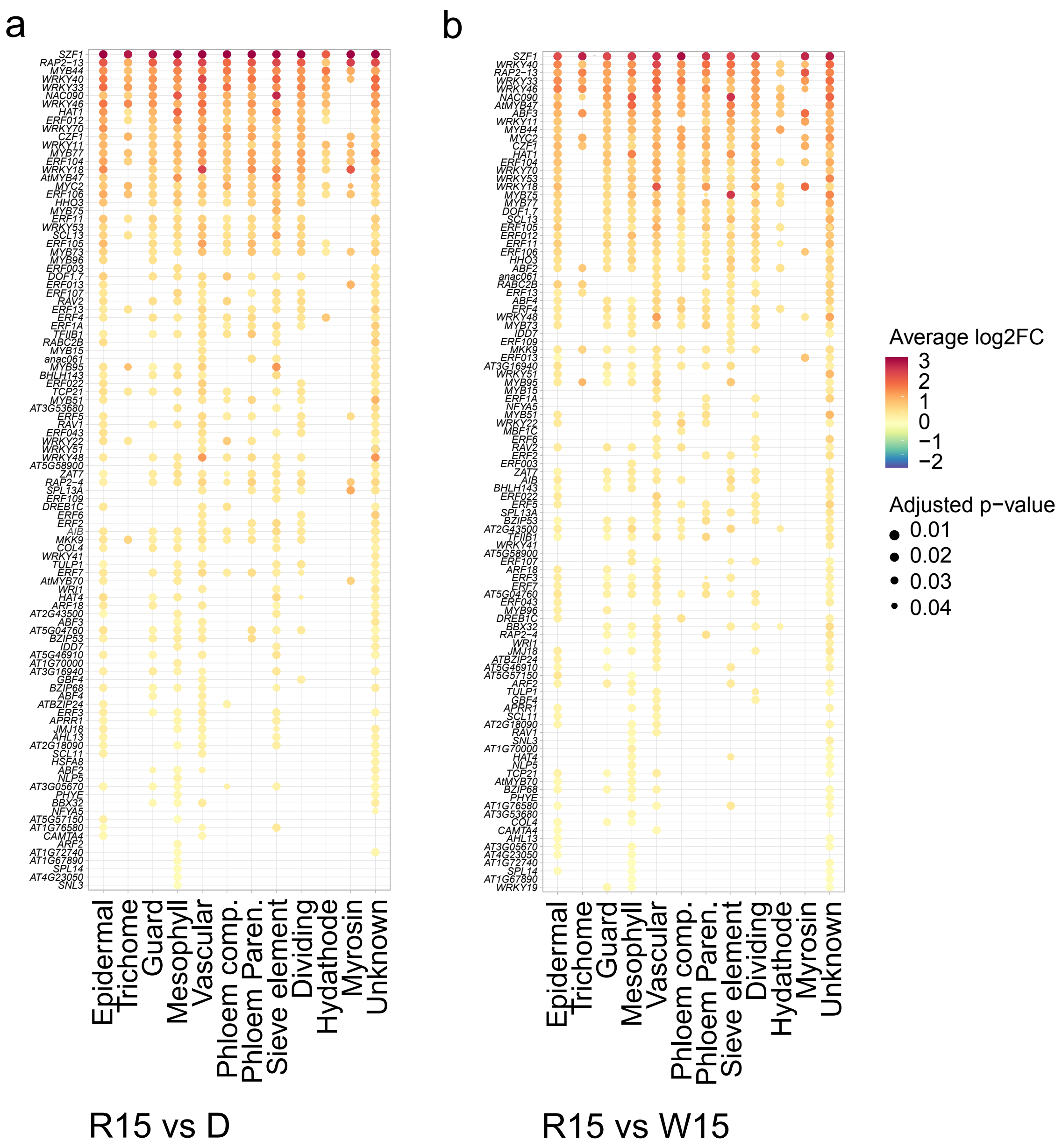

Supplementary Figure 15. TFs globally enriched after 15 minutes of rehydration across cell types.

a and b, TFs enriched globally across most cell types upon recovery (subcluster 1 in Fig. 3d), compared to
a, drought-treated cells and
b, well-watered 15-minute control cells.

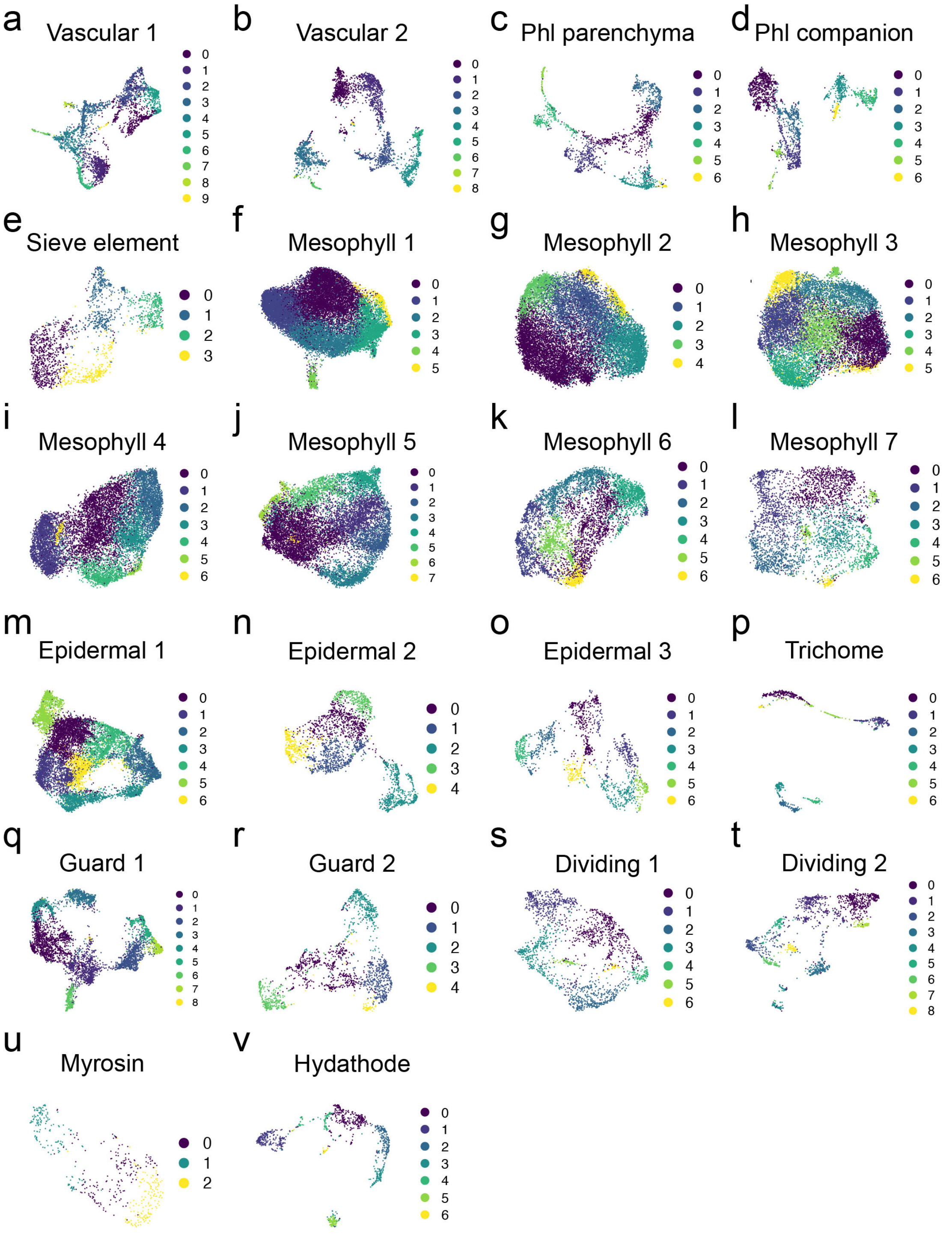

Supplementary Figure 16. Unsupervised subclustering analysis of major Arabidopsis cell types reveals internal heterogeneity.

UMAP projections of subclustered cells within major annotated cell type classes. Each panel (a–v) corresponds to one of 23 primary cell type clusters identified in the dataset, with colors indicating transcriptionally distinct subclusters identified by unsupervised clustering.
a–b, *Vascular 1–2*,
c–d, *Phloem parenchyma* and *Phloem companion cells*,
e, *Sieve element*,
f–l, *Mesophyll 1–7*,
m–o, *Epidermal 1–3*,
p, *Trichome*,

q–r, *Guard cells 1–2*,
s–t, *Dividing cells 1–2*,
u, *Myrosin*,
v, *Hydathode*
Color bars represent individual subclusters (e.g., 0 to 9), highlighting the transcriptional diversity within each major cell type. This analysis reveals specialized subpopulations and potential state transitions that may reflect functional diversity or responses to environmental stimuli.

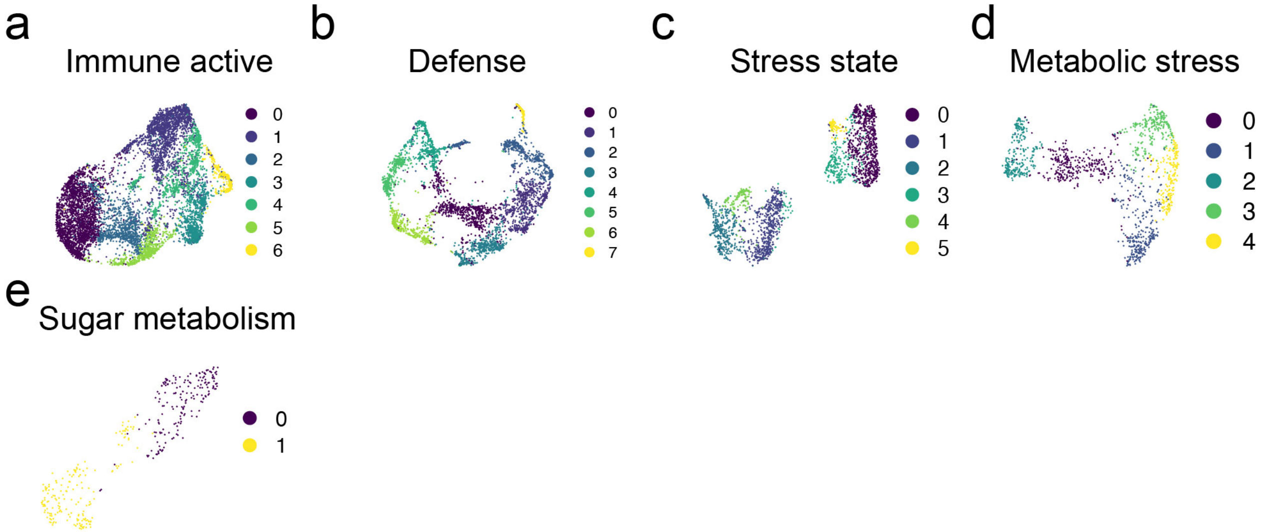

Supplementary Figure 17. Subclustering of transcriptionally distinct unknown cell populations.

UMAP projections depict subcluster identities within five transcriptionally uncharacterized cell populations.
a–e, Subclusters within:
a, associated with immune activity;
b, related to defense responses;
c, reflecting stress states;
d, linked to metabolic stress;
e, associated with sugar metabolism.

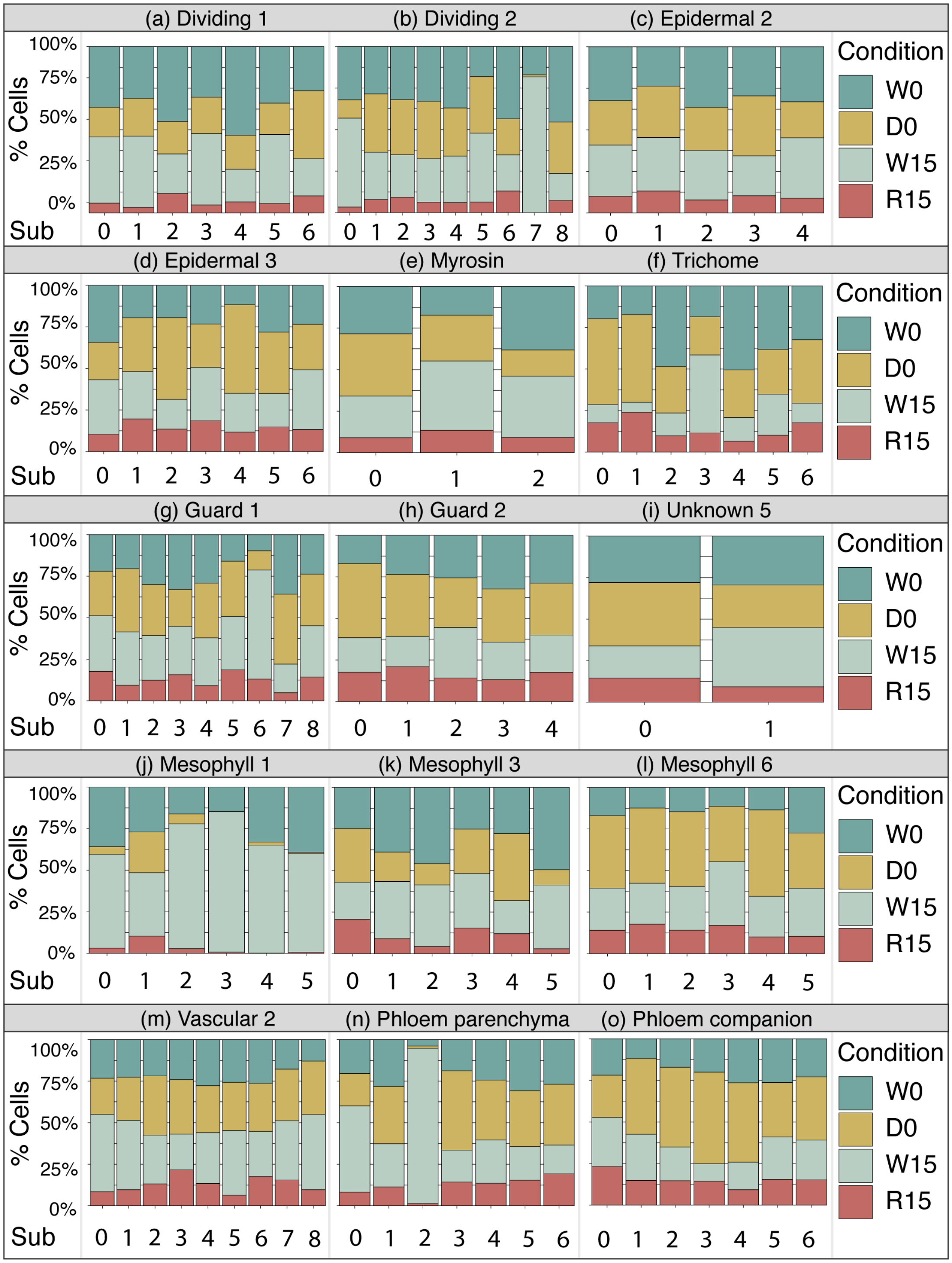

Supplementary Figure 18. The distribution of cells by condition in subclusters of major cell identity clusters.

The bar plots present cell percentage in
a, Dividing cells - 1
b, Dividing cells - 2
c, Epidermal cells - 2
d, Epidermal cells – 3
e, Myrosin cells
f, Trichome cells
g, Guard cells 1
h, Guard cells 2

i, Unknown 5
j, Mesophyll 1
k, Mesophyll 3
l, Mesophyll 6
m, Vascular 2
n, Phloem parenchyma
o, Phloem companion**.**

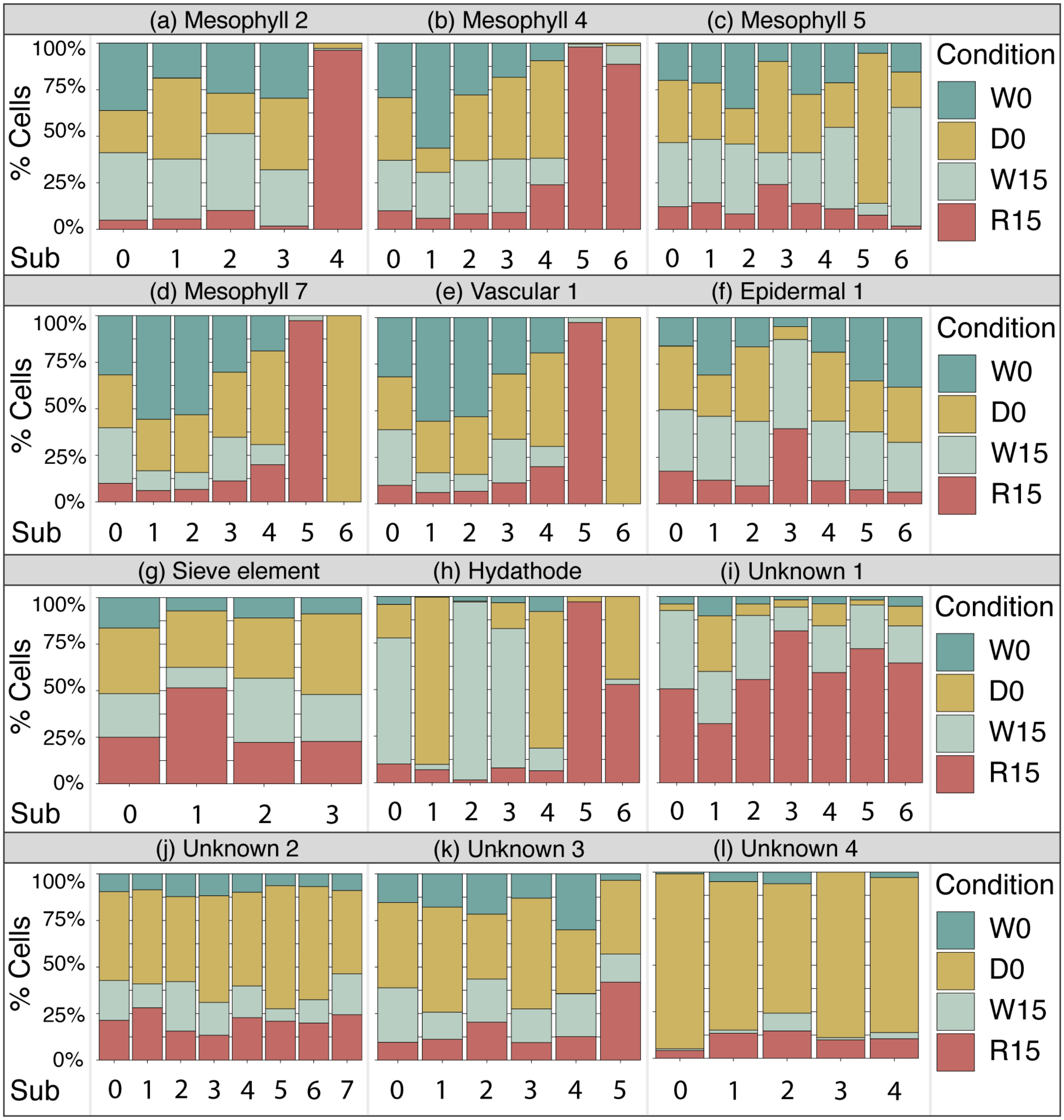

Supplementary Figure 19. The distribution of cells by condition in subclusters of major cell identity clusters.

The bar plots present cell percentage in
a, Mesophyll 2
b, Mesophyll 4
c, Mesophyll 5

d, Mesophyll 7
e, Vascular 1
f, Epidermal 1
g, Sieve element
h, Hydathode
i, Unknown 1
j, Unknown 2
k, Unknown 3
l, Unknown 4.

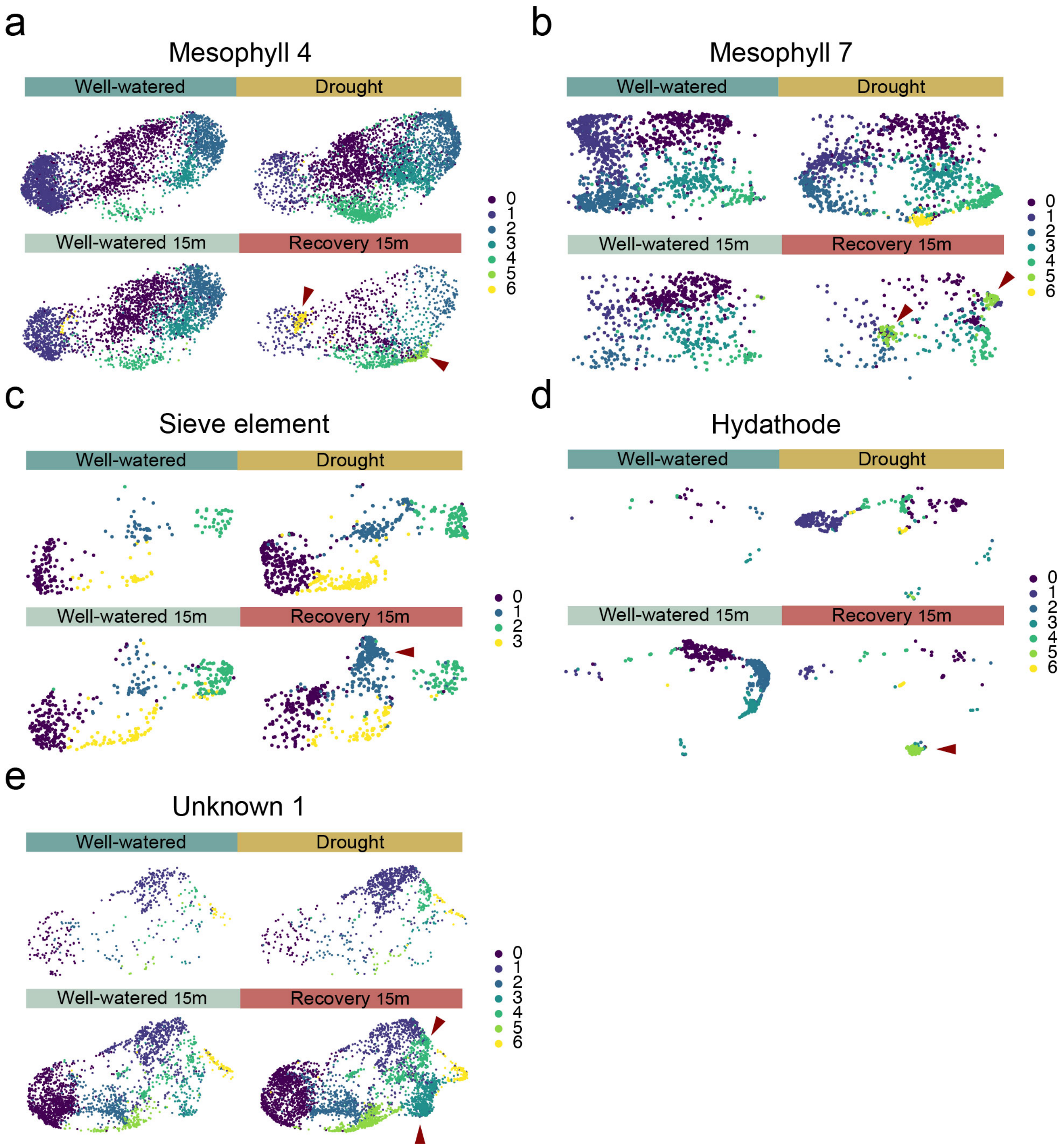

Supplementary Figure 20. Drought recovery triggers unique transcriptional state transitions in specific cell types.

a–e, UMAP projections showing subcluster transcriptional states for select cell types and an unknown cluster across four water conditions: well-watered, drought, 15 minutes after watering from well-watered (W15), and 15 minutes after watering from drought (R15).
a, Mesophyll 4 shows two unique subclusters emerging specifically in R15 (red arrowheads).
b, Mesophyll 7 also displays an R15-specific subcluster with distinct transcriptional identity.
c, Sieve element cells exhibit a state shift in response to rehydration (red arrowhead).
e, Unknown 1*, the immune active cells* are mostly cells from R15 samples, representing an enrichment of an immune active state upon rehydration.

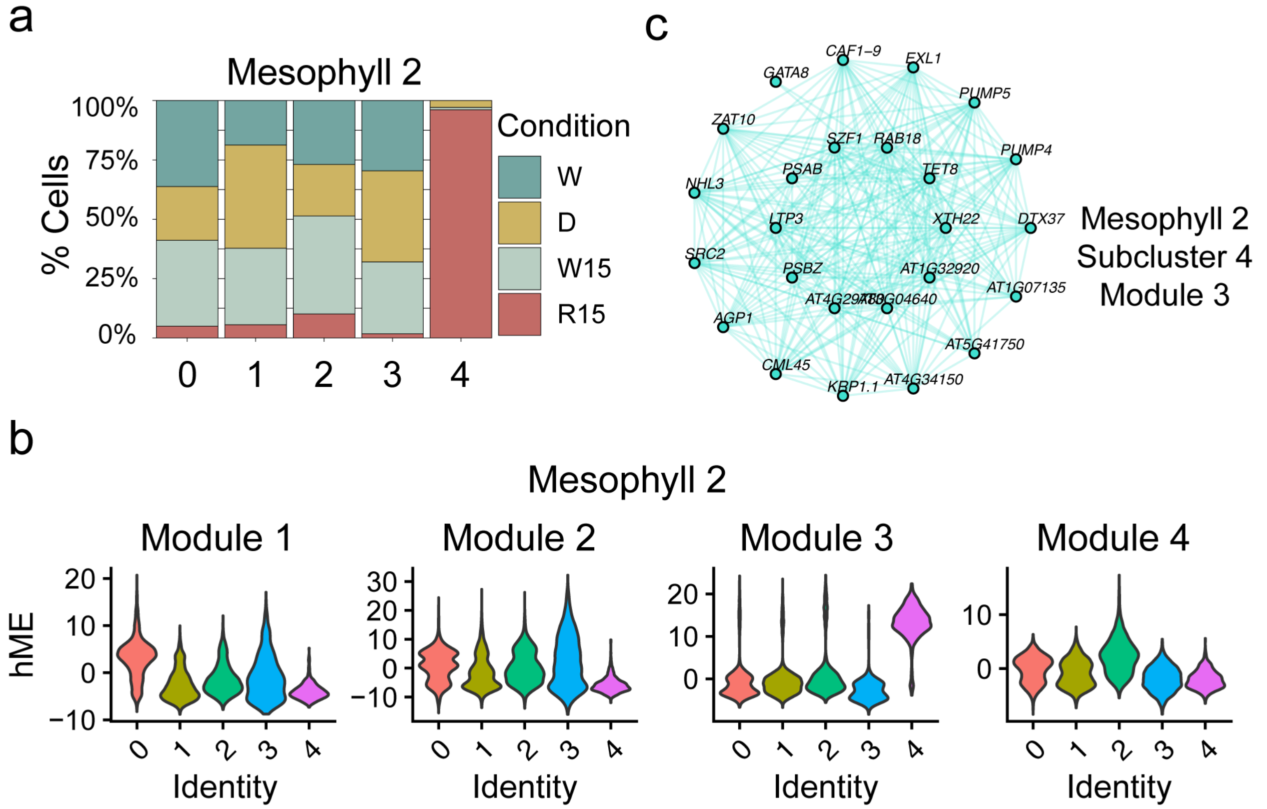

Supplementary Figure 21. Gene module identification in putative RcS.

An example of the gene network analysis performed to identify shared networks and hub genes between RcS activated in different cell types. In this example,
a, shows the subclustering of cluster "Mesophyll 2", where subcluster 4 is enriched, almost exclusively in cells from the onset of recovery.
b, Gene modules identified and their abundance in each subcluster. Module 3 is enriched in subcluster 4.
c, Top 25 hub genes of module 3. Each cell type subclusters enriched in recovery cells (over 50% of all cells in the subcluster), were used for the analysis, and hub genes were used for downstream analyses.

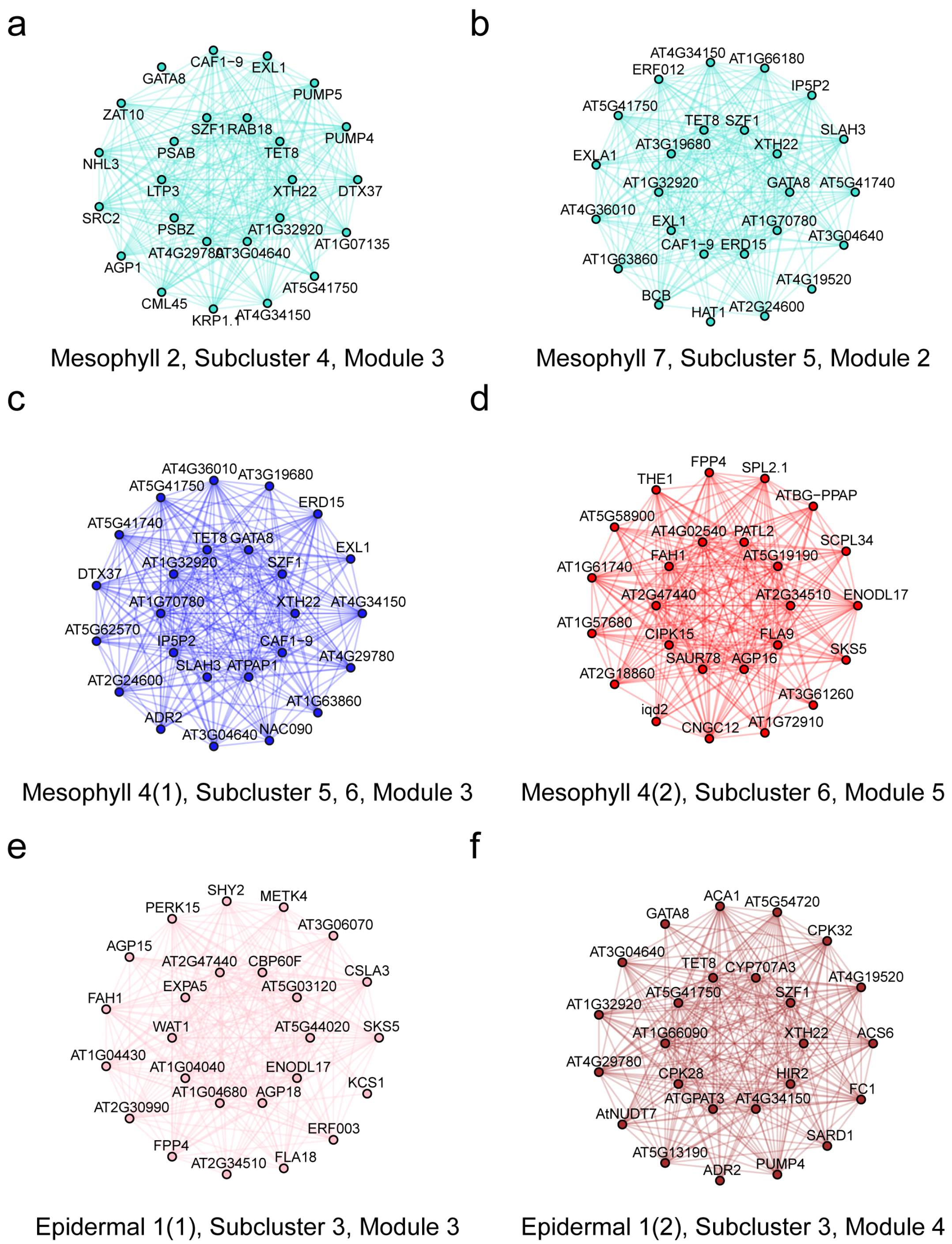

Supplementary Figure 22. Top 25 hub genes of the gene modules enriched in each RcS.

It should be noted that if there are two subclusters enriched with recovery cells in the same cell type cluster, the algorithm will not allow them to have overlapping hub genes.

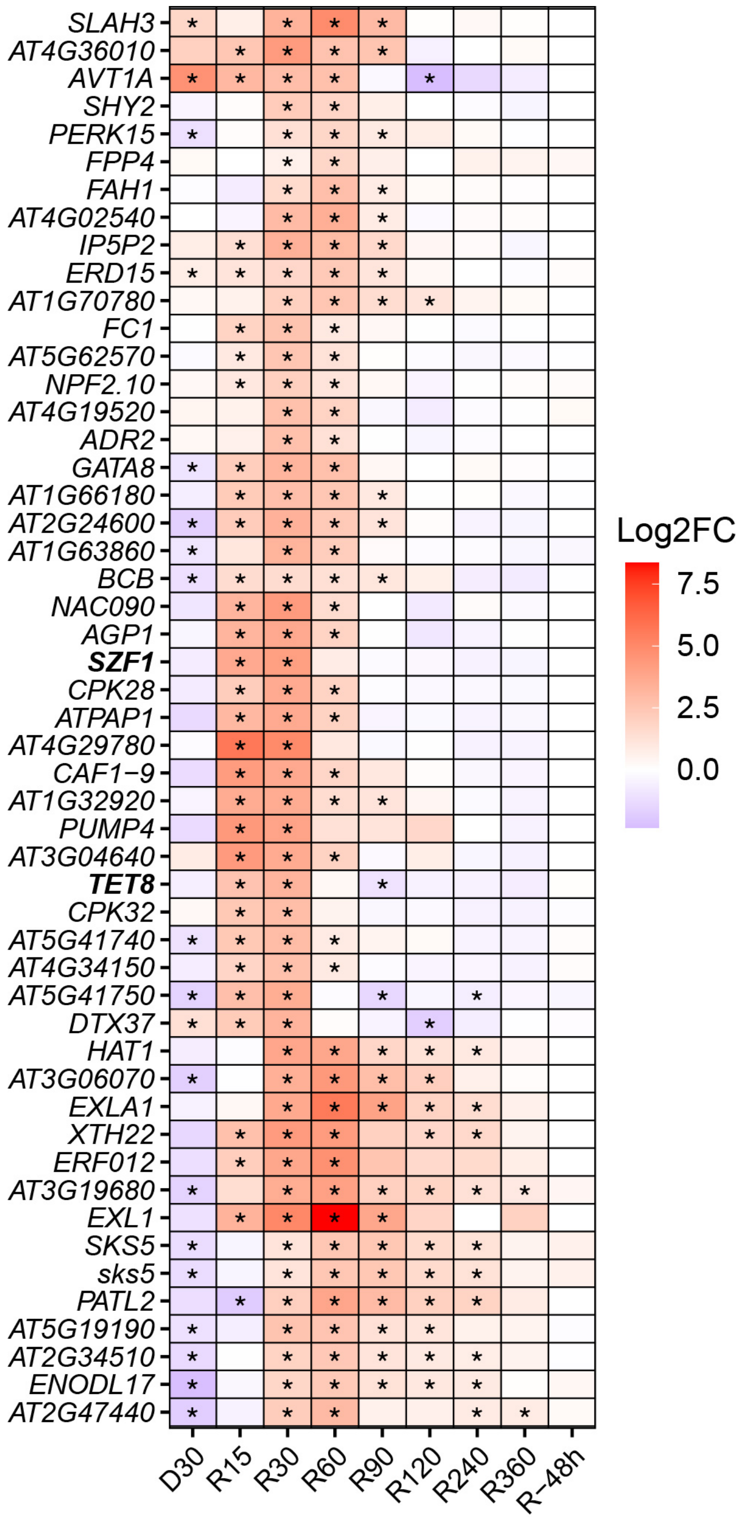

Supplementary Figure 23. RcS hub genes are also identified in our bulk drought recovery time-course.

Expression dynamics of RcS hub genes identified from snRNA-seq, shown across a bulk RNA-seq time course of drought stress and recovery. Heatmap displays log2 fold-change (log2FC) relative to the well-watered condition. Asterisks indicate statistically significant changes in expression (adjusted $P < 0.05$).

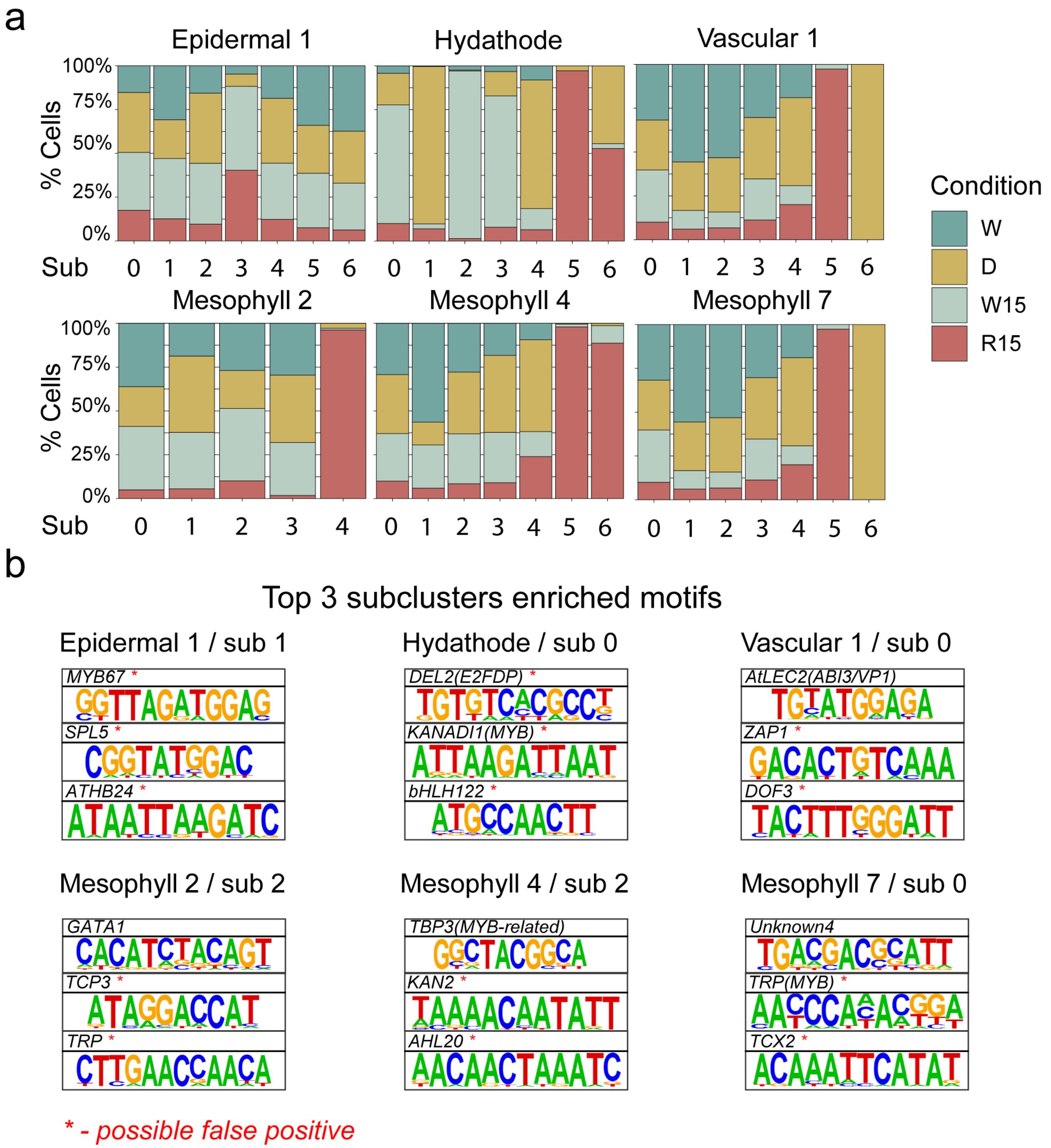

Supplementary Figure 24. Motif enrichment analysis of subclusters with equal representation of cells by condition do not show enrichment in CAMTA binding motifs.

a, Bar plots exhibit the cell percentage in each cell identity/ subcluster. The motif enrichment analysis was performed on these subclusters: Epidermal 1 – subcluster 1, Hydathode – subcluster 0, Vascular 1 – subcluster 0, Mesophyll 2 – subcluster 2, Mesophyll 4 – subcluster 2, and Mesophyll 7 – subcluster 0.

b, *de novo* Motif enrichment analysis for each recovery enriched subcluster. The gene name above the motif is the predicated TF binding the enriched motif. The analysis was performed with HOMER, using the top 100 markers from each cluster with FDR<0.05. As opposed to all RcS subclusters that were found to be enriched in the CAMTA CGCGT binding motif, only 9 subclusters (~5%) out of 162 subclusters (non RcS subclusters), were enriched with the *CAMTA* motifs (Supplementary Table S10).

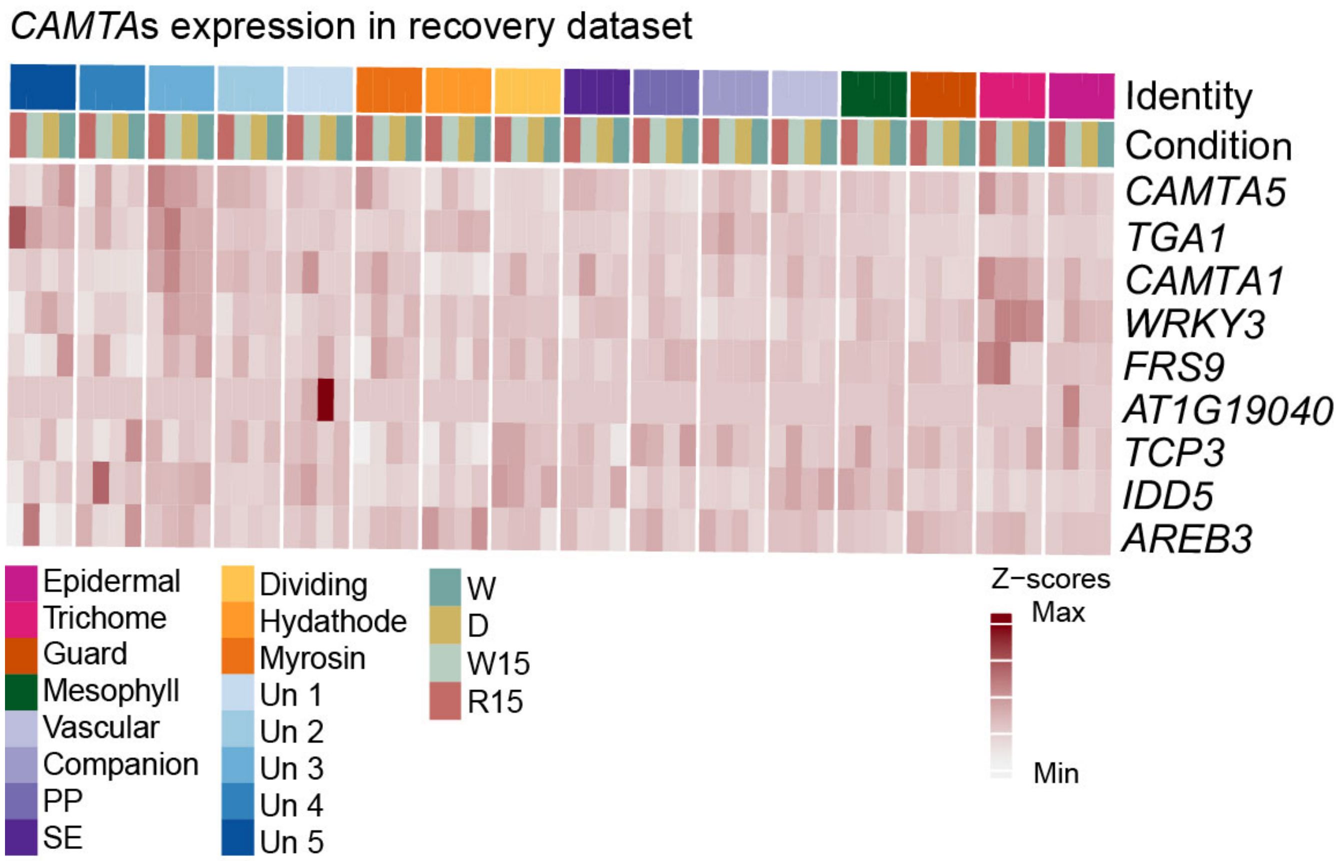

Supplementary Figure 25. The expression of *CAMTA* genes is not induced by drought recovery.

Z-score representation showing the expression levels of the TFs that putatively regulate the formation of the post-drought RcS.

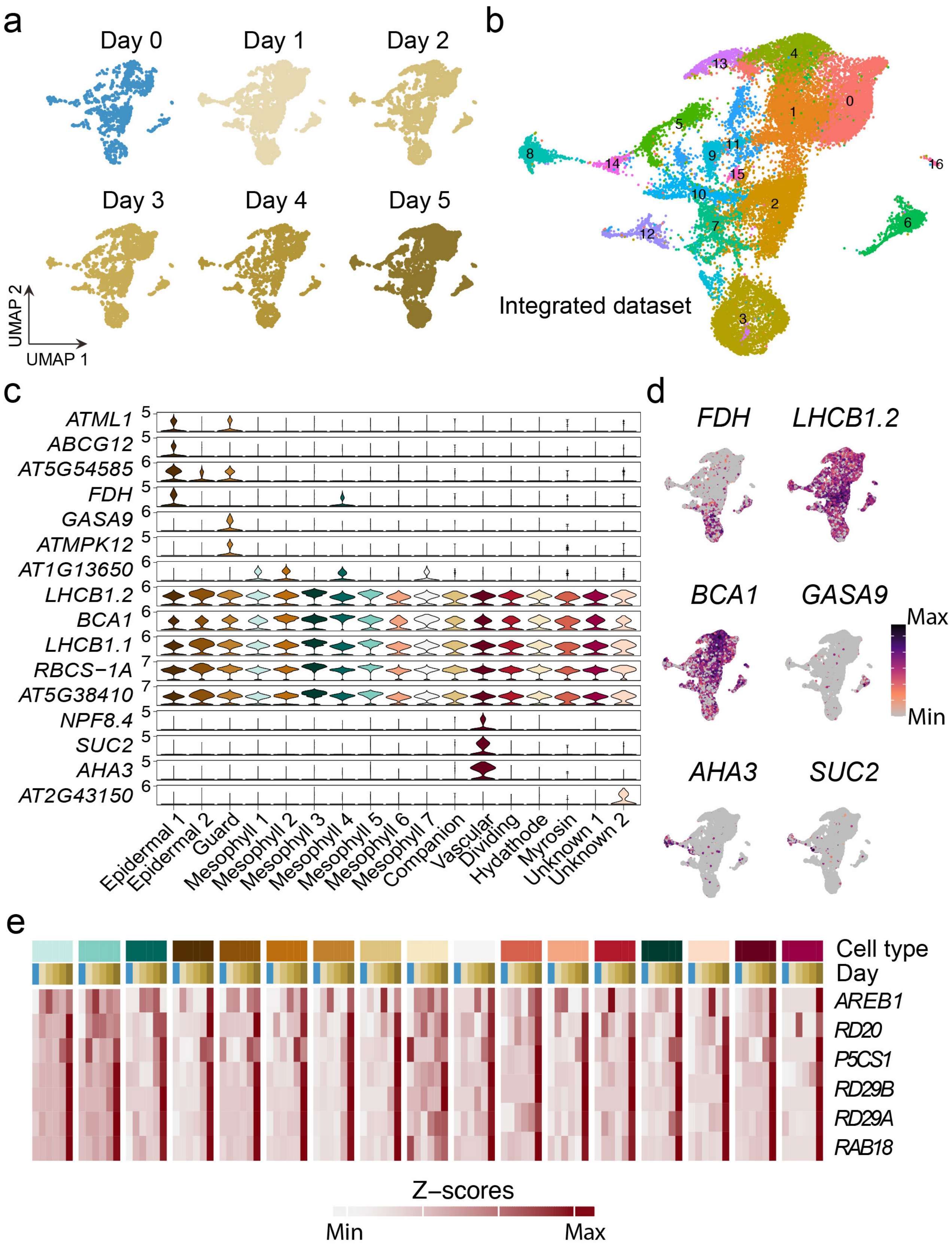

Supplementary Figure 26. snRNA-seq of plant in early stages of dehydration.

a, UMAP projection of 5 days of dehydration from a well-watered state (Day 0).
b, Seurat clusters of the integrated dataset combining all samples.
c, Violin plots presenting the expression of genes in different clusters of the integrated dataset.
d, Example literature tissue- and cell-type specific marker genes projected on our integrated UMAP.
e, The expression levels of drought inducible genes in the drought snRNA-seq dataset.

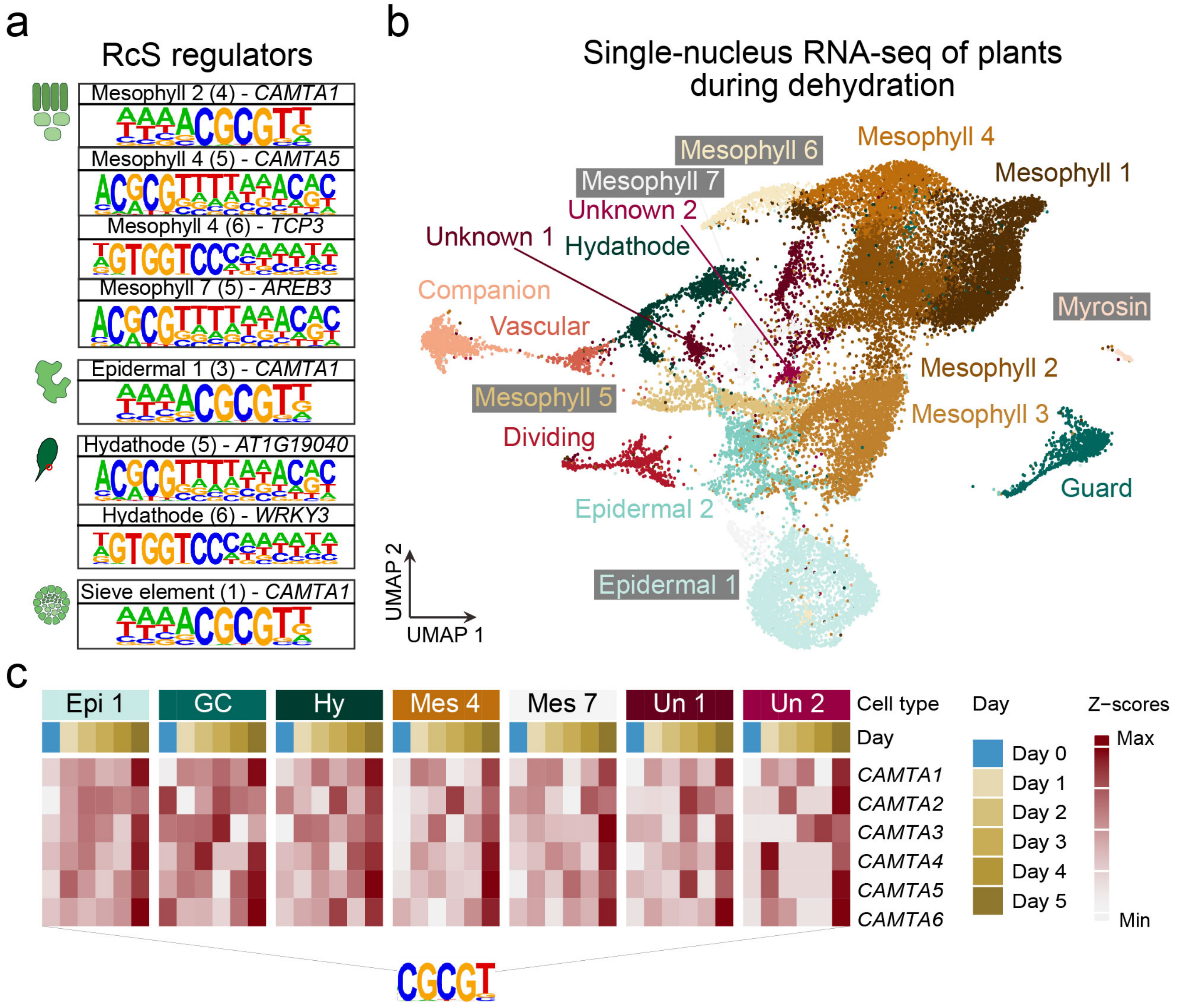

Figure 27. Potential RcS transcriptional regulators and their expression during early drought stress.

a, Motif enrichment analysis reveals candidate transcription factors likely driving the Recovery Cell State (RcS) across diverse cell types. Key motifs and predicted regulators include *CAMTA1* and *CAMTA5* in mesophyll clusters, *TCP3* and *AREB3* in stress-associated mesophyll states, *WRKY3* and *AT1G19040* in hydathodes, and *CAMTA1* in epidermal and sieve element clusters. Icons denote the corresponding cell types.
b, UMAP projection of single-nucleus transcriptomes from Arabidopsis rosettes **during early dehydration**, showing major cell types and mesophyll subclusters (1–7).
c, Heatmap showing Z-score normalized expression of *CAMTA* family members (*CAMTA1*–*CAMTA6*) across select cell types over a five-day dehydration time course. The canonical CAMTA-binding motif (CCGCGT) is shown below.

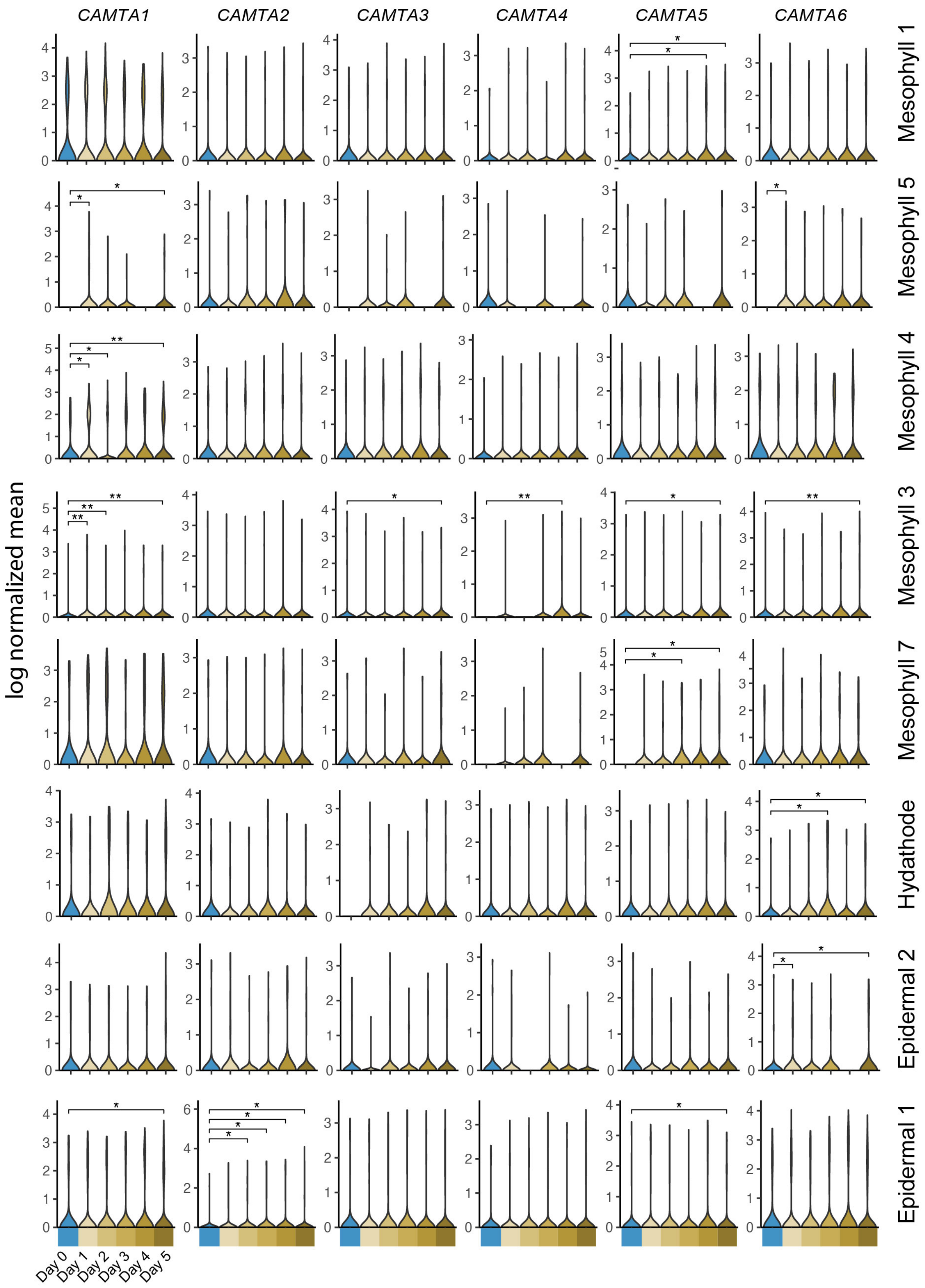

Supplementary Figure 28. The expression of CAMTA TFs in early drought stages.

Log normalized values of the expression levels of the predicted CAMTA TFs during early stages of plants dehydration in the drought dataset. Significance presented is Wilcoxon rank-sum test. It should be noted that if the Wilcoxon test is not significant, it suggests that the observed mean difference may be driven by a few high-expressing cells or may not reflect a consistent shift across the population.

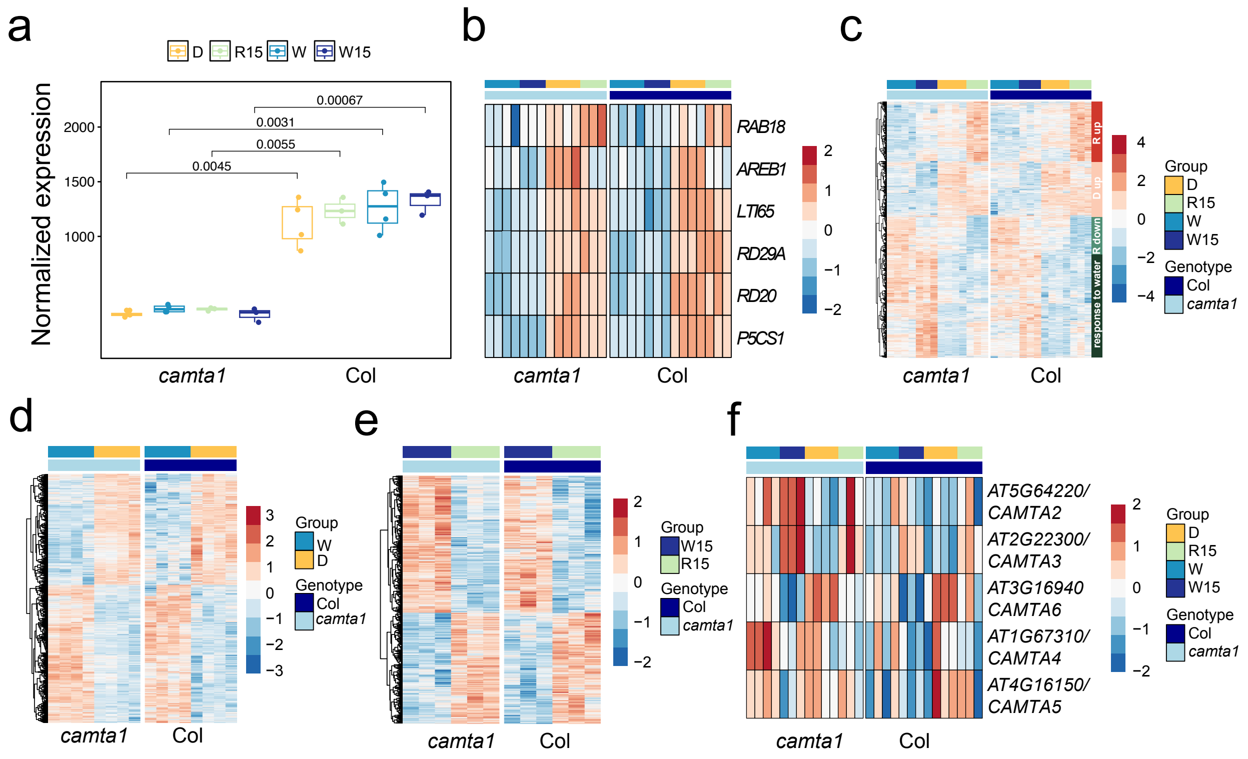

Supplementary Figure 29. *CAMTA1* does not solely regulate drought recovery.

a, Normalized expression of *CAMTA1* (*AT5G09410*) in the *camta1* mutant (SALK_008187.38.30.x), and WT (Col-0).
b, Gene expression of drought and osmotic stress marker genes in all treatments.
c, A heatmap depicting gene expression changes caused by drought, water addition, or recovery.
d, DEGs during long-term moderate drought.
e, DEGs after 15 mins of rehydration of long-term moderate drought.
f, Gene expression of other CAMTA family members. Color bar represents the Z-scores of the normalized counts.

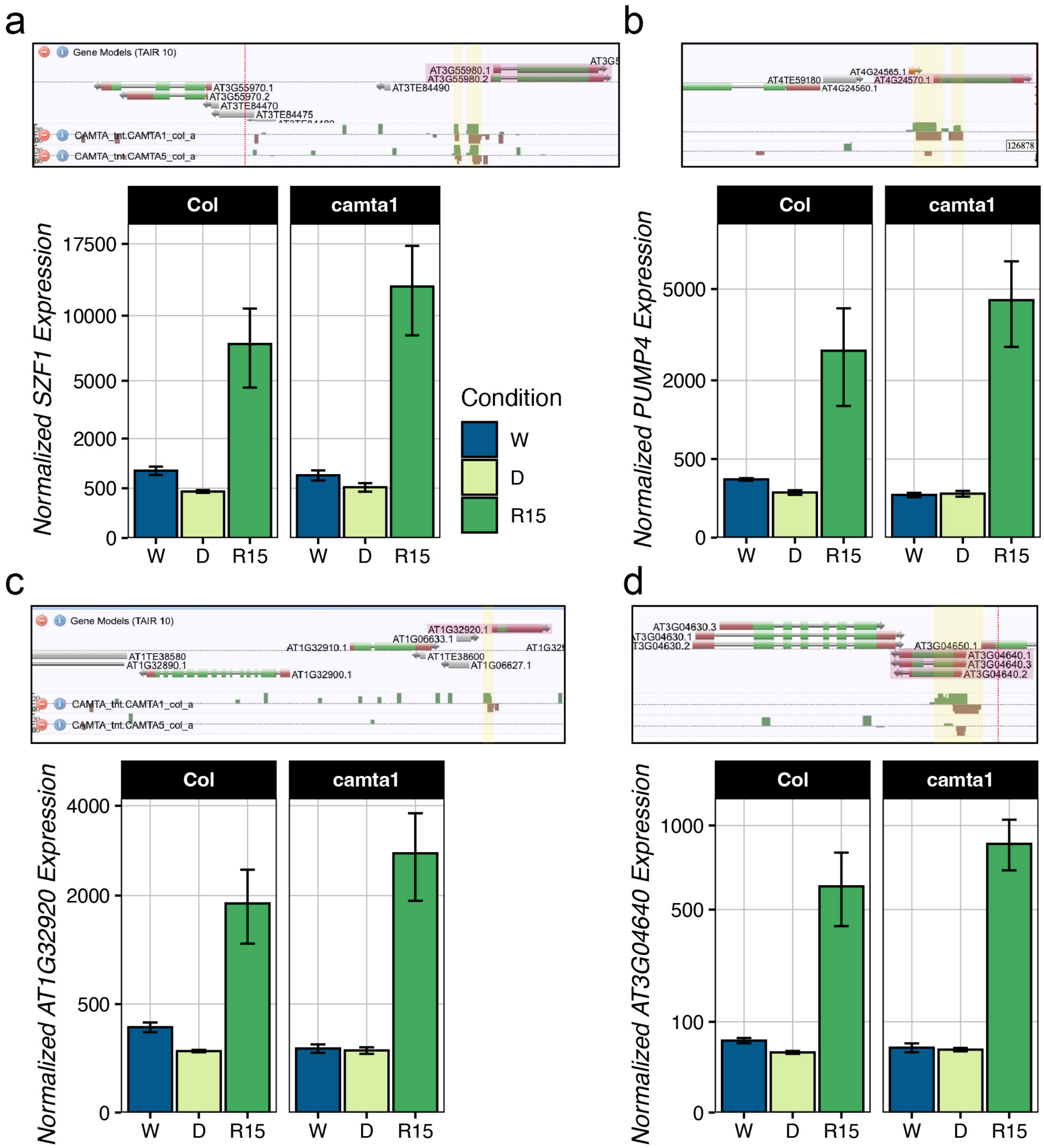

Supplementary Figure 30. Differential expression of RcS hub genes in Col-0 and *camta1* mutants under well-watered drought, and recovery conditions.

a-d, Genome browser views (top panels) show DAP-seq peak coverage and annotated gene models (TAIR10) for four RcS hub genes (genes of interest are highlighted in red). Below each genome browser track, bar plots (bottom panels) display normalized gene expression values under three conditions: well-watered (W, blue), drought (D, light green), and recovery after 15 minutes (R15, green). Error bars represent standard error of the mean (SE) from biological 3-4 replicates.
a, *SZF1* (*AT3G55980*)
b, *PUMP4* (*AT4G24570*)
c, *AT1G32920*
d, *AT3G04640*

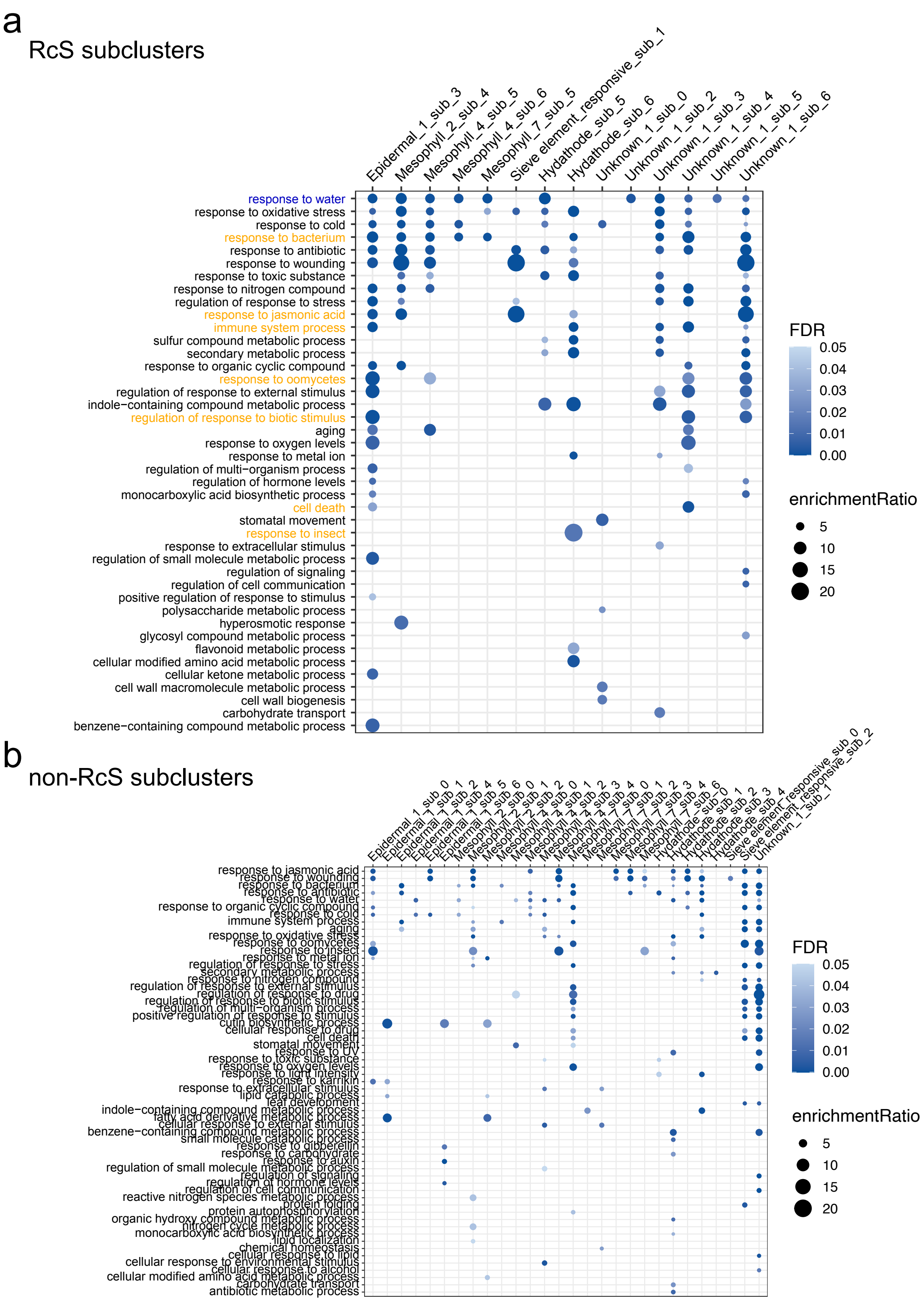

Supplementary Figure 31. Gene Ontology (GO) enrichment analysis of subclusters enriched with recovery cells (RcS) and non-RcS.

GO enrichment analysis performed on
a, RcS versus
b, non-RcS subclusters, depicting the most abundant transcriptional processes that take place during the initiation of drought recovery and the transition to the recovery cell state.

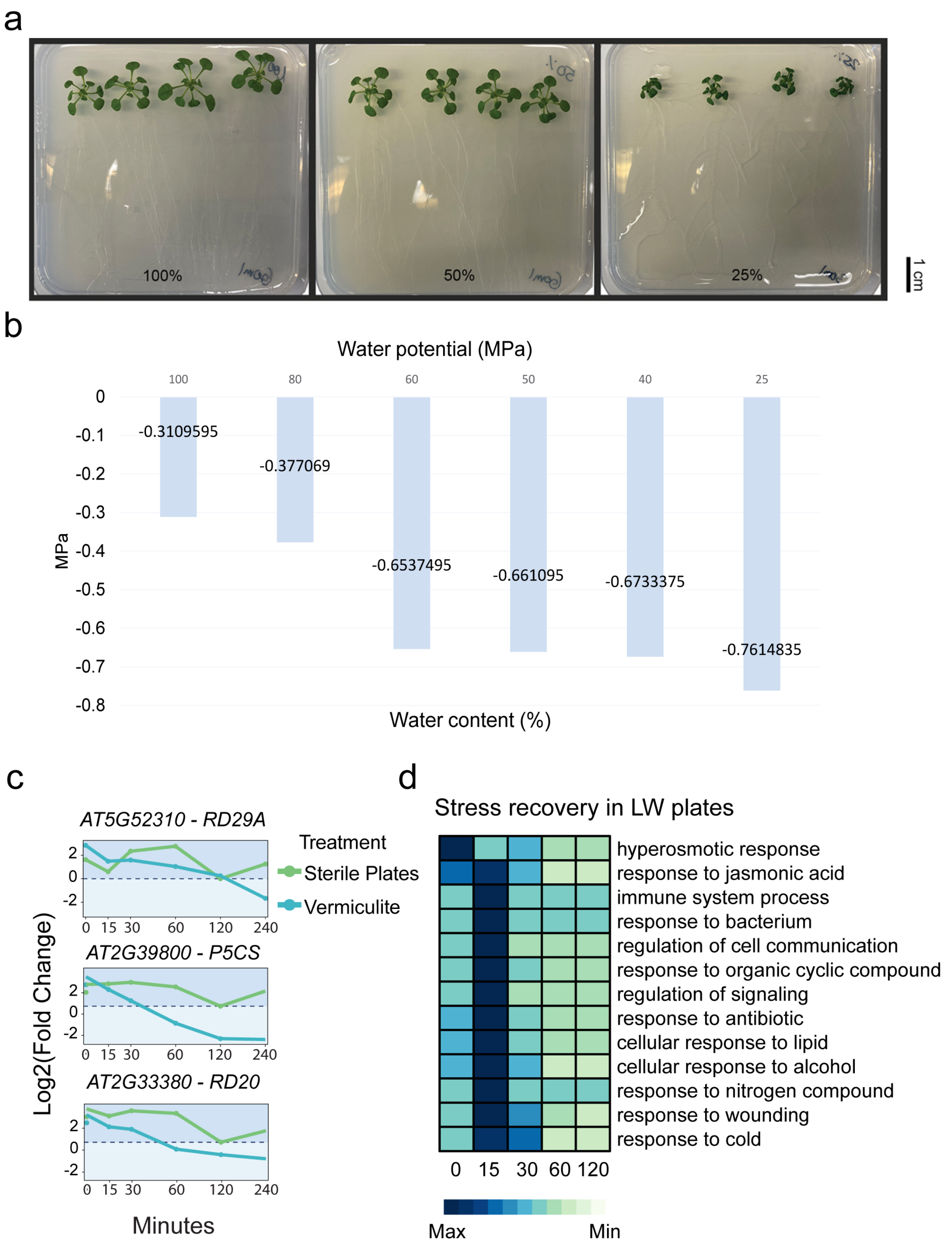

Supplementary Figure 32. LW plate system for simulating drought stress in a sterile environment.

a, plant phenotype on low water (LW) plates.
b, water potential values of LW plates.
c, Expression of drought marker genes: *RD29A*, *P5CS* and *RD20*.
d, GO enrichment analysis for genes up-regulated by moderate stress (time 0) and recovery in the low water (LW) sterile plates.

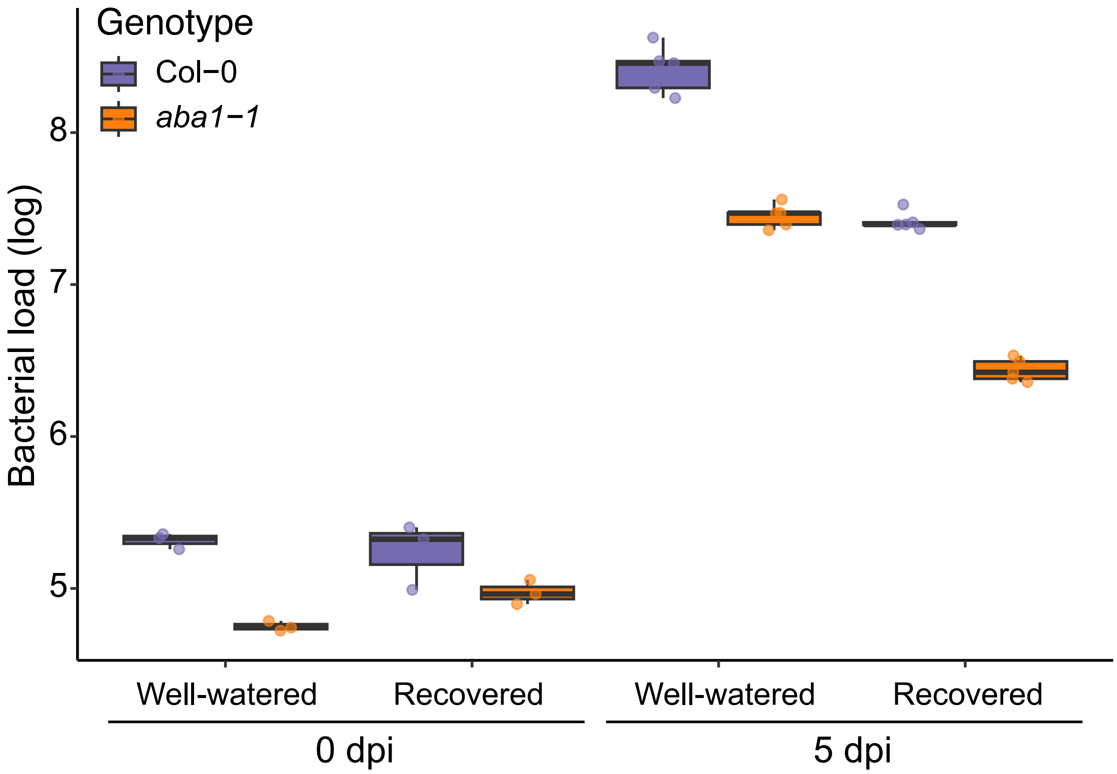

Supplementary Figure 33. Drought recovery-induced immunity in Arabidopsis is ABA independent.

Arabidopsis leaf discs were collected using a 5 mm diameter punch from leaves number 5, 6, 7, 8, and 9 at 5 days post-inoculation (dpi). For each genotype and condition combination, n = 3 for 0 dpi and n = 5 for 5 dpi.

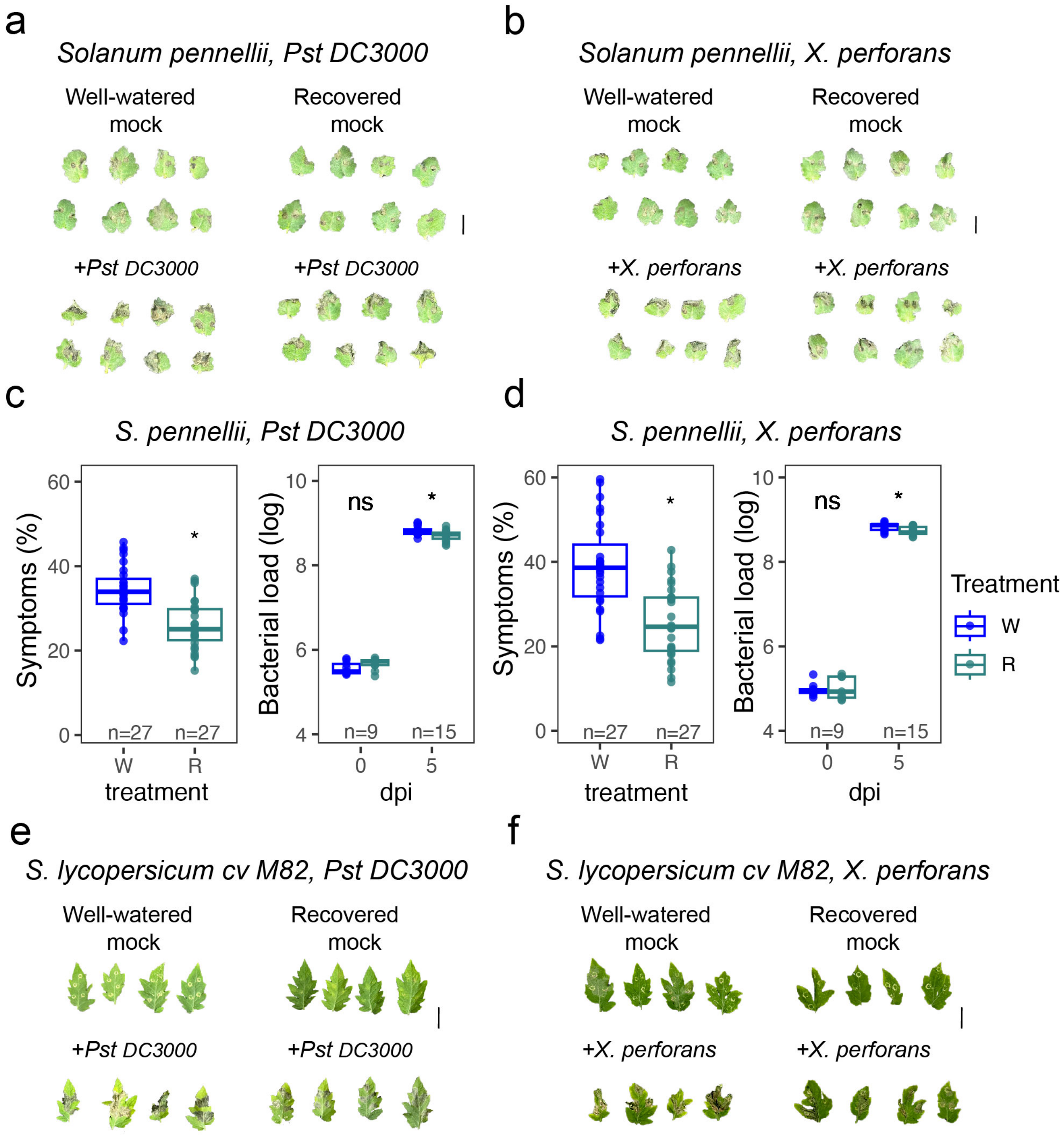

Supplementary Figure 34. Drought recovery-induced immunity enhanced resistance to *X. perforans* and *Pst* DC3000 in wild (*Solanum pennellii*) and domesticated (*S. lycopersicum* cv. M82) tomato species.

a, Representative images of well-watered or drought-recovered *S. pennellii* leaves infected with one of *Pst* DC3000, and **b**, *X. perforans*, or a mock solution. Scale bar = 1 cm.
c, Lesion percentage and bacterial load analysis of *S. pennellii* leaves five days after being syringe infiltrated with *Pst* DC3000 OD_{600}= 0.02. Inoculation was performed after 90 mins of recovery from moderate drought, and drought-treated samples were compared to well-watered controls. Results are shown for three independent experiments. Bacterial load (log_{10}CFU) of the *S. pennellii* leaves measured five days post-infection. Results are shown for three independent experiments. Two leaf discs (0.5 cm in diameter) were prepared for each sample.
d, Lesion percentage and bacterial load analysis of *S. pennellii* leaves five days after being syringe infiltrated with *X. perforans* OD_{600}= 0.02. Inoculation was performed after 90 mins of recovery from moderate drought, and drought-treated samples were compared to well-watered controls. Results are shown for three independent experiments. Bacterial load (log_{10}CFU) of the *S. pennellii* leaves measured five days post-infection. Results are shown for three independent experiments. Two leaf discs (0.5 cm in diameter) were prepared for each sample.

e, Representative images of M82 leaves infected with *Pst* DC3000, and
f, *X. perforans*, or a mock solution. Scale bar = 2 cm. Significant differences in all panels were identified using student's t-test (ns = P > 0.05, * = P ≤ 0.05, ** = P ≤ 0.01, *** = P ≤ 0.001, **** = P ≤ 0.0001). Boxplots middle line shows the median, the lower and upper hinges are the 25th and 75th percentile, respectively. The whiskers extend from the hinges to the most distant value within 1.5 * IQR of the hinge, where IQR is the inter-quartile range, or distance between the first and third quartiles.

Supplementary References

1. Abe, M., Takahashi, T. & Komeda, Y. Cloning and characterization of an L1 layer-specific gene in Arabidopsis thaliana. *Plant Cell Physiol.* **40**, 571–580 (1999).
2. Abe, M., Takahashi, T. & Komeda, Y. Identification of a cis-regulatory element for L1 layer-specific gene expression, which is targeted by an L1-specific homeodomain protein. *Plant J.* **26**, 487–494 (2001).
3. Alvarez-Buylla, E. R. *et al.* MADS-box gene evolution beyond flowers: Expression in pollen, endosperm, guard cells, roots and trichomes. *Plant J.* **24**, 457–466 (2000).
4. Baima, S. *et al.* The expression of the Athb-8 homeobox gene is restricted to provascular cells in Arabidopsis thaliana. *Development* **121**, 4171–4182 (1995).
5. Kang, J. & Dengler, N. Vein pattern development in adult leaves of Arabidopsis thaliana. *Int. J. Plant Sci.* **165**, 231–242 (2004).
6. Scarpella, E., Francis, P. & Berleth, T. Stage-specific markers define early steps of procambium development in Arabidopsis leaves and correlate termination of vein formation with mesophyll differentiation. *Development* **131**, 3445–3455 (2004).
7. Kim, J. Y. *et al.* Distinct identities of leaf phloem cells revealed by single cell transcriptomics. *Plant Cell* **33**, 511–530 (2021).
8. Barth, C. & Jander, G. Arabidopsis myrosinases TGG1 and TGG2 have redundant function in glucosinolate breakdown and insect defense. *Plant J.* **46**, 549–562 (2006).
9. Bonke, M., Thitamadee, S., Mähönen, A. P., Hauser, M. T. & Helariutta, Y. APL regulates vascular tissue identity in Arabidopsis. *Nature* **426**, 181–186 (2003).
10. Truernit, E. & Sauer, N. The promoter of the Arabidopsis thaliana SUC2 sucrose-H+ symporter gene directs expression of β-glucuronidase to the phloem: Evidence for phloem loading and unloading by SUC2. *Planta An Int. J. Plant Biol.* **196**, 564–570 (1995).
11. Bulankova, P., Akimcheva, S., Fellner, N. & Riha, K. Identification of Arabidopsis meiotic cyclins reveals functional diversification among plant cyclin genes. *PLoS Genet.* **9**(5), e1003508 (2013).
12. Chauvin, A., Caldelari, D., Wolfender, J. L. & Farmer, E. E. Four 13-lipoxygenases contribute to rapid jasmonate synthesis in wounded Arabidopsis thaliana leaves: A role for lipoxygenase 6 in responses to long-distance wound signals. *New Phytol.* **197**, 566–575 (2013).
13. Nguyen, C. T., Kurenda, A., Stolz, S., Chételat, A. & Farmer, E. E. Identification of cell populations necessary for leaf-to-leaf electrical signaling in a wounded plant. *Proc. Natl. Acad. Sci. U. S. A.* **115**, 10178–10183 (2018).
14. Chen, L. Q. *et al.* Sucrose efflux mediated by SWEET proteins as a key step for phloem transport. *Science.* **335**, 207–211 (2012).
15. Cui, H., Kong, D., Liu, X. & Hao, Y. SCARECROW, SCR-LIKE 23 and SHORT-ROOT control bundle sheath cell fate and function in Arabidopsis thaliana. *Plant J.* **78**, 319–327 (2014).
16. De Rybel, B. *et al.* A bHLH complex controls embryonic vascular tissue establishment and indeterminate growth in Arabidopsis. *Dev. Cell* **24**, 426–437 (2013).
17. Endo, M., Shimizu, H., Nohales, M. A., Araki, T. & Kay, S. A. Tissue-specific clocks in Arabidopsis show

asymmetric coupling. *Nature* **515**, 419–422 (2014).

18. Fisher, K. & Turner, S. PXY, a receptor-like kinase essential for maintaining polarity during plant vascular-tissue development. *Curr. Biol.* **17**, 1061–1066 (2007).
19. Funk, V., Kositsup, B., Zhao, C. & Beers, E. P. The Arabidopsis xylem peptidase XCP1 is a tracheary element vacuolar protein that may be a papain ortholog. *Plant Physiol.* **128**, 84–94 (2002).
20. Glaring, M. A. *et al.* An extra-plastidial α-glucan, water dikinase from Arabidopsis phosphorylates amylopectin in vitro and is not necessary for transient starch degradation. *J. Exp. Bot.* **58**, 3949–3960 (2007).
21. Gotor, C., Cejudo, F. J., Barroso, C. & Vega, J. M. Tissue-specific expression of ATCYS-3A, a gene encoding the cytosolic isoform of O-acetylserine(thiol)lyase in Arabidopsis. *Plant J.* **11**, 347–352 (1997).
22. Guo, W. J., Bundithya, W. & Goldsbrough, P. B. Characterization of the Arabidopsis metallothionein gene family: Tissue-specific expression and induction during senescence and in response to copper. *New Phytol.* **159**, 369–381 (2003).
23. Hsu, P. K. & Tsay, Y. F. Two phloem nitrate transporters, NRT1.11 and NRT1.12, are important for redistributing xylem-borne nitrate to enhance plant growth. *Plant Physiol.* **163**, 844–856 (2013).
24. Kondo, T. *et al.* Stomatal density is controlled by a mesophyll-derived signaling molecule. *Plant Cell Physiol.* **51**, 1–8 (2010).
25. Sugano, S. S. *et al.* Stomagen positively regulates stomatal density in Arabidopsis. *Nature* **463**, 241–244 (2010).
26. Lopez-Anido, C. B. *et al.* Single-cell resolution of lineage trajectories in the Arabidopsis stomatal lineage and developing leaf. *Dev. Cell* **56**, 1043-1055 (2021).
27. Johnson, C. S., Kolevski, B. & Smyth, D. R. Transparent Testa Glabra2, a trichome and seed coat development gene of arabidopsis, encodes a WRKY transcription factor. *Plant Cell* **14**, 1359–1375 (2002).
28. Kang, B. H., Busse, J. S. & Bednarek, S. Y. Members of the arabidopsis dynamin-like gene family, ADL1, are essential for plant cytokinesis and polarized cell growth. *Plant Cell* **15**, 899–913 (2003).
29. Kasahara, R. D., Portereiko, M. F., Sandaklie-Nikolova, L., Rabiger, D. S. & Drews, G. N. MYB98 is required for pollen tube guidance and synergid cell differentiation in Arabidopsis. *Plant Cell* **17**, 2981–2992 (2005).
30. Wenzel, C. L., Schuetz, M., Yu, Q. & Mattsson, J. Dynamics of MONOPTEROS and PIN-FORMED1 expression during leaf vein pattern formation in Arabidopsis thaliana. *Plant J.* **49**, 387–398 (2007).
31. Jammes, F. *et al.* MAP kinases MPK9 and MPK12 are preferentially expressed in guard cells and positively regulate ROS-mediated ABA signaling. *Proc. Natl. Acad. Sci. U. S. A.* **106**, 20520–20525 (2009).
32. Ji, J. *et al.* Wox4 promotes procambial development. *Plant Physiol.* **152**, 1346–1356 (2010).
33. Zhang, T. Q., Chen, Y. & Wang, J. W. A single-cell analysis of the Arabidopsis vegetative shoot apex. *Dev. Cell* **56**, 1056-1074.e8 (2021).
34. Procko, C. *et al.* Leaf cell-specific and single-cell transcriptional profiling reveals a role for the palisade layer in UV light protection. *Plant Cell* **34**, 3261–3279 (2022).
35. Kirschner, S. *et al.* Expression of SULTR2;2, encoding a low-affinity sulphur transporter, in the Arabidopsis bundle sheath and vein cells is mediated by a positive regulator. *J. Exp. Bot.* **69**, 4897–4906 (2018).
36. Kurata, T. *et al.* The YORE-YORE gene regulates multiple aspects of epidermal cell differentiation in Arabidopsis. *Plant J.* **36**, 55–66 (2003).

37. Mathur, J. *et al.* Transcription of the Arabidopsis CPD gene, encoding a steroidogenic cytochrome P450, is negatively controlled by brassinosteroids. *Plant J.* **14**, 593–602 (1998).
38. Müller, R., Borghi, L., Kwiatkowska, D., Laufs, P. & Simon, R. Dynamic and compensatory responses of Arabidopsis shoot and floral to meristems to CLV3 signaling. *Plant Cell* **18**, 1188–1198 (2006).
39. Chen, L. Q. *et al.* Sugar transporters for intercellular exchange and nutrition of pathogens. *Nature* **468**, 527–532 (2010).
40. Mustroph, A. *et al.* Profiling translatomes of discrete cell populations resolves altered cellular priorities during hypoxia in Arabidopsis. *Proc. Natl. Acad. Sci. U. S. A.* **106**, 18843–18848 (2009).
41. Zhang, L. *et al.* Altered xylem-phloem transfer of amino acids affects metabolism and leads to increased seed yield and oil content in Arabidopsis. *Plant Cell* **22**, 3603–3620 (2010).
42. Ohashi-Ito, K. & Bergmann, D. C. Arabidopsis FAMA controls the final proliferation/differentiation switch during stomatal development. *Plant Cell* **18**, 2493–2505 (2006).
43. Okumoto, S. *et al.* High affinity amino acid transporters specifically expressed in xylem parenchyma and developing seeds of Arabidopsis. *J. Biol. Chem.* **277**, 45338–45346 (2002).
44. Pilot, G. *et al.* Overexpression of GLUTAMINE DUMPER1 leads to hypersecretion of glutamine from hydathodes of arabidopsis leaves. *Plant Cell* **16**, 1827–1840 (2004).
45. Pommerrenig, B. *et al.* Phloem-Specific expression of Yang cycle genes and identification of novel Yang cycle enzymes in Plantago and Arabidopsis. *Plant Cell* **23**, 1904–1919 (2011).
46. Qiu, J. L., Jilk, R., Marks, M. D. & Szymanski, D. B. The Arabidopsis SPIKE1 gene is required for normal cell shape control and tissue development. *Plant Cell* **14**, 101–118 (2002).
47. Susek, R. E., Ausubel, F. M. & Chory, J. Signal transduction mutants of arabidopsis uncouple nuclear CAB and RBCS gene expression from chloroplast development. *Cell* **74**, 787–799 (1993).
48. Ranjan, A., Fiene, G., Fackendahl, P. & Hoecker, U. The Arabidopsis repressor of light signaling SPA1 acts in the phloem to regulate seedling de-etiolation, leaf expansion and flowering time. *Development* **138**, 1851–1862 (2011).
49. Stadler, R. & Sauer, N. The Arabidopsis thaliana AtSUC2 gene is specifically expressed in companion cells. *Bot. Acta* **109**, 299–306 (1996).
50. Rouse, D. T., Marotta, R. & Parish, R. W. Promoter and expression studies on an Arabidopsis thaliana dehydrin gene. *FEBS Lett.* **381**, 252–256 (1996).
51. Zimmermann, I., Saedler, R., Mutondo, M. & Hulskamp, M. The Arabidopsis GNARLED gene encodes the NAP125 homolog and controls several actin-based cell shape changes. *Mol. Genet. Genomics* **272**, 290–296 (2004).
52. Zhu, Y., Liu, L., Shen, L. & Yu, H. NaKR1 regulates long-distance movement of FLOWERING LOCUS T in Arabidopsis. *Nat. Plants* **2**, (2016).
53. Zhang, X., Dyachok, J., Krishnakumar, S., Smith, L. G. & Oppenheimer, D. G. IRREGULAR TRICHOME BRANCH1 in arabidopsis encodes a plant homolog of the actin-related protein2/3 complex activator Scar/WAVE that regulates actin and microtubule organization. *Plant Cell* **17**, 2314–2326 (2005).
54. Yoshimoto, N., Inoue, E., Saito, K., Yamaya, T. & Takahashi, H. Phloem-localizing sulfate transporter, Sultr1;3,

mediates re-distribution of sulfur from source to sink organs in arabidopsis. *Plant Physiol.* **131**, 1511–1517 (2003).

55. Yephremov, A., *et al*. Characterization of the FIDDLEHEAD gene of Arabidopsis reveals a link between adhesion response and cell differentiation in the epidermis. *Plant cell*, **11**(11), 2187–2201. (1999).
56. Xu, W. *et al.* Arabidopsis TCH4, regulated by hormones and the environment, encodes a xyloglucan endotransglycosylase. *Plant Cell* **7**, 1555–1567 (1995).
57. Wu, H. *et al.* Molecular and biochemical characterization of the Fe(III) chelate reductase gene family in Arabidopsis thaliana. *Plant Cell Physiol.* **46**, 1505–1514 (2005).
58. Werner, T. *et al.* Cytokinin-deficient transgenic Arabidopsis plants show multiple developmental alterations indicating opposite functions of cytokinins in the regulation of shoot and root meristem activity. *Plant Cell* **15**(11), 2532–2550 (2003).
59. Uemoto, K., Araki, T. & Endo, M. Isolation of arabidopsis palisade and spongy mesophyll cells. *Methods Mol. Biol.* **1830**, 141–148 (2018).
60. Takemiya, A. *et al.* Phosphorylation of BLUS1 kinase by phototropins is a primary step in stomatal opening. *Nat. Commun.* **4**, 2094. (2013).
61. Takada, S., Takada, N. & Yoshida, A. ATML1 promotes epidermal cell differentiation in Arabidopsis shoots. *Dev.* **140**, 1919–1923 (2013).
62. Szymanski, D. B., Jilk, R. A., Pollock, S. M. & Marks, M. D. Control of GL2 expression in Arabidopsis leaves and trichomes. *Development* **125**, 1161–1171 (1998).
63. Zhang, F., Gonzalez, A., Zhao, M., Payne, C. T. & Lloyd, A. A network of redundant bHLH proteins functions in all TTG1-dependent pathways of Arabidopsis. *Development* **130**, 4859–4869 (2003).
64. Xia, Y., Nikolau, B. J. & Schnable, P. S. Developmental and hormonal regulation of the Arabidopsis CER2 gene that codes for a nuclear-localized protein required for the normal accumulation of cuticular waxes. *Plant Physiol.* **115**, 925–937 (1997).
65. Wallner, E. S. *et al.* Strigolactone- and Karrikin-Independent SMXL Proteins Are Central Regulators of Phloem Formation. *Curr. Biol.* **27**(8), 1241–1247 (2017).
66. Walker, A. R. *et al.* The TRANSPARENT TESTA GLABRA1 locus, which regulates trichome differentiation and anthocyanin biosynthesis in arabidopsis, encodes a WD40 repeat protein. *Plant Cell* **11**, 1337–1349 (1999).
67. Vanzin, G. F. *et al.* The mur2 mutant of Arabidopsis thaliana lacks fucosylated xyloglucan because of a lesion in fucosyltransferase AtFUT1. *Proc. Natl. Acad. Sci. U. S. A.* **99**, 3340–3345 (2002).
68. Van Leene, J. *et al.* Targeted interactomics reveals a complex core cell cycle machinery in Arabidopsis thaliana. *Mol. Syst. Biol.* **6**, (2010).
69. Vahisalu, T. *et al.* SLAC1 is required for plant guard cell S-type anion channel. *Nature* **452**, 487–491 (2008).
70. Takahashi, K. *et al.* Ectopic expression of an esterase, which is a candidate for the unidentified plant cutinase, causes cuticular defects in arabidopsis thaliana. *Plant Cell Physiol.* **51**, 123–131 (2010).
71. Ohashi, Y., Oka, A., Ruberti, I., Morelli, G. & Aoyama, T. Entopically additive expression of GLABRA2 alters the frequency and spacing of trichome initiation. *Plant J.* **29**, 359–369 (2002).
72. Swaminathan, K., Yang, Y., Grotz, N., Campisi, L. & Jack, T. An enhancer trap line associated with a D-class cyclin gene in Arabidopsis. *Plant Physiol.* **124**, 1658–1667 (2000).

73. Sistrunk, M. L., Antosiewicz, D. M., Purugganan, M. M. & Braam, J. Arabidopsis TCH3 encodes a novel Ca2+ binding protein and shows environmentally induced and tissue-specific regulation. *Plant Cell* **6**, 1553–1565 (1994).

74. Sinlapadech, T., Stout, J., Ruegger, M. O., Deak, M. & Chapple, C. The hyper-fluorescent trichome phenotype of the brt1 mutant of Arabidopsis is the result of a defect in a sinapic acid:UDPG glucosyltransferase. *Plant J.* **49**, 655–668 (2007).

75. Schlereth, A. *et al.* MONOPTEROS controls embryonic root initiation by regulating a mobile transcription factor. *Nature* **464**, 913–916 (2010).

76. Schellmann, S. *et al.* TRIPTYCHON and CAPRICE mediate lateral inhibition during trichome and root hair patterning in Arabidopsis. *EMBO J.* **21**, 5036–5046 (2002).

77. Sawchuk, M. G., Donner, T. J., Head, P. & Scarpella, E. Unique and overlapping expression patterns among members of photosynthesis-associated nuclear gene families in Arabidopsis. *Plant Physiol.* **148**, 1908–1924 (2008).

78. Sawa, S. *et al.* The HAT2 gene, a member of the HD-Zip gene family, isolated as an auxin inducible gene by DNA microarray screening, affects auxin response in Arabidopsis. *Plant J.* **32**, 1011–1022 (2002).

79. Saedler, R., Zimmermann, I., Mutondo, M. & Hülskamp, M. The Arabidopsis KLUNKER gene controls cell shape changes and encodes the AtSRA1 homolog. *Plant Mol. Biol.* **56**, 775–782 (2004).

80. Endo, M., Mochizuki, N., Suzuki, T. & Nagatani, A. CRYPTOCHROME2 in vascular bundles regulates flowering in Arabidopsis. *Plant Cell* **19**, 84–93 (2007).

81. Redovniković, I. R., Textor, S., Lisnić, B. & Gershenzon, J. Expression pattern of the glucosinolate side chain biosynthetic genes MAM1 and MAM3 of Arabidopsis thaliana in different organs and developmental stages. *Plant Physiol. Biochem.* **53**, 77–83 (2012).

82. Pilot, G., Gaymard, F., Mouline, K., Chérel, I. & Sentenac, H. Regulated expression of Arabidopsis Shaker K+ channel genes involved in K+ uptake and distribution in the plant. *Plant Mol. Biol.* **51**, 773–787 (2003).

83. Oppenheimer, D. G. *et al.* Essential role of a kinesin-like protein in Arabidopsis trichome morphogenesis. *Proc. Natl. Acad. Sci. U. S. A.* **94**, 6261–6266 (1997).

84. Kirik, V. *et al.* Ectopic expression of the Arabidopsis AtMYB23 gene induces differentiation of trichome cells. *Dev. Biol.* **235**, 366–377 (2001).

85. Dinkeloo, K., Boyd, S. & Pilot, G. Update on amino acid transporter functions and on possible amino acid sensing mechanisms in plants. *Semin. Cell Dev. Biol.* **74**, 105–113 (2018).

86. Negi, J. *et al.* A dof transcription factor, SCAP1, is essential for the development of functional stomata in arabidopsis. *Curr. Biol.* **23**, 479–484 (2013).

87. Geisler, M., Nadeau, J. & Sack, F. D. Oriented asymmetric divisions that generate the stomatal spacing pattern in arabidopsis are disrupted by the too many mouths mutation. *Plant Cell* **12**, 2075–2086 (2000).

88. Weichert, A. *et al.* AtPTR4 and AtPTR6 are differentially expressed, tonoplast-localized members of the peptide transporter/nitrate transporter 1 (PTR/NRT1) family. *Planta* **235**, 311–323 (2012).

89. Schachtman, D. P. *et al.* Expression of an inward-rectifying potassium channel by the Arabidopsis KAT1 cDNA. *Science (New York, N.Y.).* **258**, 5088 (1992).

90. Menges, M. & Murray, J. A. H. Synchronous Arabidopsis suspension cultures for analysis of cell-cycle gene activity. *Plant J.* **30**, 203–212 (2002).
91. Mayer, K F et al. Role of WUSCHEL in regulating stem cell fate in the Arabidopsis shoot meristem. *Cell*. **95**(6), 805-815. (1998).
92. MacAlister, C. A., Ohashi-Ito, K. & Bergmann, D. C. Transcription factor control of asymmetric cell divisions that establish the stomatal lineage. *Nature* **445**, 537–540 (2007).
93. Pillitteri, L. J., Sloan, D. B., Bogenschutz, N. L. & Torii, K. U. Termination of asymmetric cell division and differentiation of stomata. *Nature* **445**, 501–505 (2007).
94. Schuster, J., Knill, T., Reichelt, M., Gershenzon, J. & Binder, S. BRANCHED-CHAIN AMINOTRANSFERASE4 is part of the chain elongation pathway in the biosynthesis of methionine-derived glucosinolates in Arabidopsis. *Plant Cell* **18**, 2664–2679 (2006).
95. Lu, L., Lee, Y. R. J., Pan, R., Maloof, J. N. & Liu, B. An internal motor kinesin is associated with the golgi apparatus and plays a role in trichome morphogenesis in Arabidopsis. *Mol. Biol. Cell* **16**, 811–823 (2005).
96. Li, S. F., Higginson, T. & Parish, R. W. A novel MYB-related gene from Arabidopsis thaliana expressed in developing anthers. *Plant Cell Physiol.* **40**, 343–347 (1999).
97. Lai, L. B. *et al.* The Arabidopsis R2R3 MYB proteins FOUR LIPS and MYB88 restrict divisions late in the stomatal cell lineage. *Plant Cell* **17**, 2754–2767 (2005).
98. Kubo, M. *et al.* Transcription switches for protoxylem and metaxylem vessel formation. *Genes Dev*. **19**(16), 1855-1860. (2005)
99. Kirik, V. *et al.* Functional diversification of MYB23 and GL1 genes in trichome morphogenesis and initiation. *Development* **132**, 1477–1485 (2005).
100. O'Malley, R. C. *et al*. Cistrome and epicistrome features shape the regulatory DNA landscape. *Cell.* **165(**5), 1280-1292. (2016).
101. Bar-Peled, M. & O'Neill, M. A. Plant nucleotide sugar formation, interconversion, and salvage by sugar recycling. *Annu Rev Plant Biol*. **62**,127-155. (2011).
102. Lalonde, S., Wipf, D. & Frommer, W. B. Transport mechanisms for organic forms of carbon and nitrogen between source and sink. *Annu Rev Plant Biol*. **55**, 341-372. (2004).
103. Cobbett, C. & Goldsbrough, P. Phytochelatins and metallothioneins: roles in heavy metal detoxification and homeostasis. *Annu Rev Plant Biol*. **53**, 159-182. (2002).
104. Vong, G.Y.W., McCarthy, K., Claydon, W., Davis, S.J., Redmond, E.J. & Ezer ,D. AraLeTA: An Arabidopsis leaf expression atlas across diurnal and developmental scales. *Plant Physiol*. **195**(3), 1941-1953. (2024).
105. Berkowitz, O., Xu, Y., Liew, L.C. *et al*. RNA-seq analysis of laser microdissected Arabidopsis thaliana leaf epidermis, mesophyll and vasculature defines tissue-specific transcriptional responses to multiple stress treatments. *Plant J*. **107**(3), 938-955. (2021).
106. Delannoy, E., Batardiere, B., Pateyron, S., *et al*. Cell specialization and coordination in Arabidopsis leaves upon pathogenic attack revealed by scRNA-seq. *Plant Commun*. **4**(5), 100676. (2023).
