# Supplementary material for: Drought recovery in plants triggers a cell-state-specific immune activation
Source: Nat Commun. 2025 Aug 29;16:8095. doi: 10.1038/s41467-025-63467-2 (PMC12397292; doi:10.1038/s41467-025-63467-2)
Supplement: Supplementary file 2 — Description of Additional Supplementary Files [file 41467_2025_63467_MOESM2_ESM.pdf]

Description of Additional Supplementary Files

Supplementary Data 1

Drought-Responsive Genes - DE under drought, values are the average of three replicates, Log2FC. D= DROUGHT, R=RECOVERY.

Supplementary Data 2

Recovery Specific Genes, values are the average of three replicates, log2FC. D= DROUGHT, R=RECOVERY.

Supplementary Data 3

Single-nucleus data summary.

Supplementary Data 4

Literature known cell-type or tissue specific marker genes used for annotation.

Supplementary Data 5

Single nucleus RNA-seq top 50 cluster marker genes.

Supplementary Data 6

Novel cell type marker genes

Supplementary Data 7

Arabidopsis TFs induced in all cell-types after 15 mins of post-drought rehydration. Below: statistical analysis of DE subcluster 1 R15 vs W15.

Supplementary Data 8

Arabidopsis TFs induced specifically in dividing cells after 15 mins of post-drught rehydration.

Supplementary Data 9

Hub genes found in gene modules enriched in recovery cell-states.

Supplementary Data 10

Motif enrichment analysis for all subclusters.
