# Supplementary material for: Drought recovery in plants triggers a cell-state-specific immune activation
Source: Nat Commun. 2025 Aug 29;16:8095. doi: 10.1038/s41467-025-63467-2 (PMC12397292; doi:10.1038/s41467-025-63467-2)
Supplement: Supplementary file 4 — Reporting Summary [file 41467_2025_63467_MOESM4_ESM.pdf]

nature portfolio

Corresponding author(s): Joseph R. Ecker

Last updated by author(s): 08/01/2025

Reporting Summary

Nature Portfolio wishes to improve the reproducibility of the work that we publish. This form provides structure for consistency and transparency in reporting. For further information on Nature Portfolio policies, see our Editorial Policies and the Editorial Policy Checklist.

Statistics

For all statistical analyses, confirm that the following items are present in the figure legend, table legend, main text, or Methods section.

n/a	Confirmed	
[ ]	[x]	The exact sample size (*n*) for each experimental group/condition, given as a discrete number and unit of measurement
[ ]	[x]	A statement on whether measurements were taken from distinct samples or whether the same sample was measured repeatedly
[ ]	[x]	The statistical test(s) used AND whether they are one- or two-sided<br>*Only common tests should be described solely by name; describe more complex techniques in the Methods section.*
[ ]	[x]	A description of all covariates tested
[ ]	[x]	A description of any assumptions or corrections, such as tests of normality and adjustment for multiple comparisons
[ ]	[x]	A full description of the statistical parameters including central tendency (e.g. means) or other basic estimates (e.g. regression coefficient) AND variation (e.g. standard deviation) or associated estimates of uncertainty (e.g. confidence intervals)
[ ]	[x]	For null hypothesis testing, the test statistic (e.g. *F*, *t*, *r*) with confidence intervals, effect sizes, degrees of freedom and *P* value noted<br>*Give P values as exact values whenever suitable.*
[x]	[ ]	For Bayesian analysis, information on the choice of priors and Markov chain Monte Carlo settings
[ ]	[x]	For hierarchical and complex designs, identification of the appropriate level for tests and full reporting of outcomes
[x]	[ ]	Estimates of effect sizes (e.g. Cohen's *d*, Pearson's *r*), indicating how they were calculated

Our web collection on statistics for biologists contains articles on many of the points above.

Software and code

Policy information about availability of computer code

Data collection	All data used for analysis in our study was generated by us for this study, and all code used for data analysis in this study (both for bulk RNA-seq datasets and single nucleus RNA-seq) is described in this publicly available github page: https://github.com/NatanellaIE/DroughtRecovery
Data analysis	all code used for data analysis in this study (both for bulk RNA-seq datasets and single nucleus RNA-seq) is described in this publicly available github page: https://github.com/NatanellaIE/DroughtRecovery https://doi.org/10.5281/zenodo.16652205.

For manuscripts utilizing custom algorithms or software that are central to the research but not yet described in published literature, software must be made available to editors and reviewers. We strongly encourage code deposition in a community repository (e.g. GitHub). See the Nature Portfolio guidelines for submitting code & software for further information.

Data

Policy information about availability of data

All manuscripts must include a data availability statement. This statement should provide the following information, where applicable:

- Accession codes, unique identifiers, or web links for publicly available datasets
- A description of any restrictions on data availability
- For clinical datasets or third party data, please ensure that the statement adheres to our policy

The sequencing data generated in this study have been deposited in the NCBI database under accession code GSE220278 [https://www.ncbi.nlm.nih.gov/geo/query/acc.cgi?acc= GSE220278]. The spatial transcriptomics data can be found in the same link, series GSE303744.

Research involving human participants, their data, or biological material

Policy information about studies with human participants or human data. See also policy information about sex, gender (identity/presentation), and sexual orientation and race, ethnicity and racism.

Reporting on sex and gender	NA
Reporting on race, ethnicity, or other socially relevant groupings	NA
Population characteristics	NA
Recruitment	NA
Ethics oversight	NA

Note that full information on the approval of the study protocol must also be provided in the manuscript.

Field-specific reporting

Please select the one below that is the best fit for your research. If you are not sure, read the appropriate sections before making your selection.

[x] Life sciences [ ] Behavioural & social sciences [ ] Ecological, evolutionary & environmental sciences

For a reference copy of the document with all sections, see nature.com/documents/nr-reporting-summary-flat.pdf

Life sciences study design

All studies must disclose on these points even when the disclosure is negative.

Sample size	Sample size depends on the experiments we used, for bulk RNA-seq, each condition * time point had at least 3 biological replicates collected and processed for the analysis. For single-nucleus RNA-seq, 30-100 of Arabidopsis rosettes were collected and snapped-freezen in liquid nitrogen. Drought recovery samples had 2 biological replicates of multiple rosettes and nuclei processed separately, and two technical replicates of these two biological replicates.
Data exclusions	In the stomatal conductance analysis (Fig. 2b) 1 out of 4 replicates was an outlier that was removed, resulting with 3 biological replicates. The outlier is shown in the source data file and is colored red.
Replication	Drought recovery samples had 2 biological replicates of multiple rosettes and nuclei processed separately, and two technical replicates of these two biological replicates.
Randomization	In mutants experiments (camta1-ko), WT and mutant plants were placed randomly on trays within the growth chambers used, to eliminate a location effect within the chamber/ tray.
Blinding	NA

Reporting for specific materials, systems and methods

We require information from authors about some types of materials, experimental systems and methods used in many studies. Here, indicate whether each material, system or method listed is relevant to your study. If you are not sure if a list item applies to your research, read the appropriate section before selecting a response.

Materials & experimental systems

n/a	Involved in the study	
[x]	[ ]	Antibodies
[x]	[ ]	Eukaryotic cell lines
[x]	[ ]	Palaeontology and archaeology
[x]	[ ]	Animals and other organisms
[x]	[ ]	Clinical data
[x]	[ ]	Dual use research of concern
[ ]	[x]	Plants

Methods

n/a	Involved in the study	
[x]	[ ]	ChIP-seq
[x]	[ ]	Flow cytometry
[x]	[ ]	MRI-based neuroimaging

Dual use research of concern

Policy information about dual use research of concern

Hazards

Could the accidental, deliberate or reckless misuse of agents or technologies generated in the work, or the application of information presented in the manuscript, pose a threat to:

No	Yes	
☒	☐	Public health
☒	☐	National security
☒	☐	Crops and/or livestock
☒	☐	Ecosystems
☒	☐	Any other significant area

Experiments of concern

Does the work involve any of these experiments of concern:

No	Yes	
☒	☐	Demonstrate how to render a vaccine ineffective
☒	☐	Confer resistance to therapeutically useful antibiotics or antiviral agents
☒	☐	Enhance the virulence of a pathogen or render a nonpathogen virulent
☒	☐	Increase transmissibility of a pathogen
☒	☐	Alter the host range of a pathogen
☒	☐	Enable evasion of diagnostic/detection modalities
☒	☐	Enable the weaponization of a biological agent or toxin
☒	☐	Any other potentially harmful combination of experiments and agents

Plants

Seed stocks	Col-0, camta1: SALK_008187.38.30.x, aba1-1: EMS mutant first described in Koornneef et al. (1982).
Novel plant genotypes	NA
Authentication	PCR was performed to validate the insertion in camta1 (SALK_008187.38.30.x).
